# Crystal structures of two mononuclear complexes of terbium(III) nitrate with the tripodal alcohol 1,1,1-tris­(hy­droxy­meth­yl)propane

**DOI:** 10.1107/S2056989017001116

**Published:** 2017-01-27

**Authors:** Thaiane Gregório, Siddhartha O. K. Giese, Giovana G. Nunes, Jaísa F. Soares, David L. Hughes

**Affiliations:** a Departamento de Química, Universidade Federal do Paraná, Centro Politécnico, Jardim das Américas, 81530-900 Curitiba-PR, Brazil; b School of Chemistry, University of East Anglia, University Plain, Norwich NR4 7TJ, UK

**Keywords:** crystal structure, lanthanide, terb­ium(III), nitrate, mononuclear, tripodal alcohol

## Abstract

Two new mononuclear cationic complexes in which the Tb^III^ ion is bis-chelated by the tripodal alcohol 1,1,1-tris­(hy­droxy­meth­yl)propane (H_3_ *L* ^Et^, C_6_H_14_O_3_) were prepared from Tb(NO_3_)_3_·5H_2_O and had their crystal and mol­ecular structures solved by single-crystal X-ray diffraction analysis after data collection at 100 K. Both products were isolated in reasonable yields from the same reaction mixture by using different crystallization conditions. The higher-symmetry complex dinitratobis[1,1,1-tris­(hy­droxy­meth­yl)propane]­terbium(III) nitrate di­meth­oxy­ethane hemisolvate, [Tb(NO_3_)_2_(H_3_ *L* ^Et^)_2_]NO_3_·0.5C_4_H_10_O_2_, **1**, in which the lanthanide ion is 10-coordinate and adopts an *s*-bicapped square-anti­prismatic coordination geometry, contains two bidentate nitrate ions bound to the metal atom; another nitrate ion functions as a counter-ion and a half-mol­ecule of di­meth­oxy­ethane (completed by a crystallographic twofold rotation axis) is also present. In product aqua­nitratobis[1,1,1-tris­(hy­droxy­meth­yl)propane]­terbium(III) dinitrate, [Tb(NO_3_)(H_3_ *L* ^Et^)_2_(H_2_O)](NO_3_)_2_, **2**, one bidentate nitrate ion and one water mol­ecule are bound to the nine-coordinate terbium(III) centre, while two free nitrate ions contribute to charge balance outside the tricapped trigonal-prismatic coordination polyhedron. No free water mol­ecule was found in either of the crystal structures and, only in the case of **1**, di­meth­oxy­ethane acts as a crystallizing solvent. In both mol­ecular structures, the two tripodal ligands are bent to one side of the coordination sphere, leaving room for the anionic and water ligands. In complex **2**, the methyl group of one of the H_3_ *L* ^Et^ ligands is disordered over two alternative orientations. Strong hydrogen bonds, both intra- and inter­molecular, are found in the crystal structures due to the number of different donor and acceptor groups present.

## Abstract 2

Two new mononuclear complexes of ten- and nine-coordinate terbium(III) were synthesized from Tb(NO_3_)_3_·5H_2_O and the tripodal alcohol 1,1,1-tris­(hy­droxy­meth­yl)propane (H_3_ *L* ^Et^) to be employed as potential building blocks for heterometallic 3*d*–4*f* block metal aggregates.

## Chemical context   

Our inter­est in developing synthetic routes for the synthesis of mono- or polynuclear complexes containing lanthanide(III) ions is based on the possibility that these compounds behave as single-ion (SIM) or single-mol­ecule (SMM) magnets (Benelli & Gatteschi, 2015 ▸; Gatteschi *et al.*, 2006 ▸; Frost *et al.*, 2016 ▸; Meng *et al.*, 2016 ▸). In such chemical species, it is usually possible to exploit the strong spin-orbit coupling, the relatively high-spin angular momentum and the large magnetic anisotropy presented by lanthanides to maximize the energy barrier for the reversal of the magnetization (Luzon & Sessoli, 2012 ▸; Vieru *et al.*, 2016 ▸; Sessoli & Powell, 2009 ▸) and therefore increase the technological applicability of these materials.

With this objective in mind, our first steps were the synthesis and characterization of complexes containing *Ln* ^III^ ions that could be used as building blocks for polynuclear 3*d*–4*f* block metal aggregates. The first report of a heterometallic complex of this type that showed SMM behaviour described the tetra­nuclear mol­ecule [{Cu^II^ *L*Tb^III^(Hfac)_2_}_2_] [H_3_ *L* = 1-(2-hy­droxy­benzamido)-2-(2-hy­droxy-3-meth­oxy-benzyl­idene­amino)­ethane and Hfac = hexa­fluoro­acetyl­acetone], obtained by self-assembly (Osa *et al.*, 2004 ▸). Magnetic studies of the product revealed ferromagnetic exchange and slow relaxation of the magnetization at low temperatures, with a potential energy barrier Δ/*kB* of 21 K (14.7 cm^−1^).

After this report, many other heterometallic complexes containing 3*d* and 4*f* ions with different structures and nuclearities were characterized as single-mol­ecule magnets (Liu *et al.*, 2015 ▸). In 2014, a trinuclear complex of dyspros­ium(III) and iron(II) presented the largest potential energy barrier reported to date for this type of system. The mol­ecule, formulated as [Fe^II^_2_Dy^III^ *L*_2_(H_2_O)]ClO_4_·2H_2_O, *L* = 2,2′,2′′-{[nitrilo­tris­(ethane-2,1-di­yl)]tris­(aza­nedi­yl)methyl­ene}tris­(4-chloro­phenol), and also synthesized in a self-assembly reaction, presents two iron(II) ions in different coordination environments (octa­hedral and distorted trigonal prismatic) bound to a dysprosium(III) ion in *quasi-D*_5*h*_ symmetry, which is pointed out by the authors as fundamental for the observed SMM behaviour and for the impressive potential energy barrier of 459 K (319 cm^−1^) (Liu *et al.*, 2014 ▸). This value, although lower than the record figures reported for lanthan­ide-containing SIM compounds (Liu *et al.*, 2016 ▸), still reveals the potential of mixed 3*d*–4*f* metal complexes to behave as quantum magnets.

Despite these good results, most of the experimental procedures employed for the preparation of these polynuclear compounds involve self-assembly reactions, which often compete with the rational design of the desired mol­ecules. Many efforts have been directed recently to the development of synthetic routes that allow for greater predictability of the formed products, both structural and with respect to their magnetic properties, employing simple and elegant experimental procedures that include modular synthesis approaches (Kahn, 1997 ▸; Stumpf *et al.*, 1993 ▸).

In this context, the present work involved reactions between the tripodal alcohol H_3_ *L* ^Et^ [1,1,1-tris­(hy­droxy­meth­yl)propane] and Tb(NO_3_)_3_·5H_2_O that generated the new, cationic complexes [Tb(H_3_ *L* ^Et^)_2_(NO_3_)_2_](NO_3_)·0.5glyme (product **1**) and [Tb(H_3_ *L* ^Et^)_2_(NO_3_)(H_2_O)](NO_3_)_2_ (product **2**). In both cases, the coordination environment of the lanthanide ion is similar to that observed in the central unit (core) of star-shaped heterometallic SMMs of general formula [*M*_3_ *M*′(*L* ^Et^)_2_(dpm)_3_] (*M* and *M*′ = transition metal(III) ions; *L* ^Et^ = EtC(CH_2_O)_3_^3−^ tripodal alkoxide and Hdpm = dipivaloyl­methane) (Accorsi *et al.*, 2006 ▸; Totaro *et al.*, 2013 ▸; Westrup *et al.*, 2014 ▸; Gregoli *et al.*, 2009 ▸). Complexes **1** and **2** were characterized by elemental and X-ray diffraction analysis, together with vibrational (infrared) spectroscopy. These products are potential building blocks to be subsequently combined, in stoichiometric proportions, with other 3*d* and 4*f* starting materials to give heterometallic products with potentially inter­esting magnetic properties.
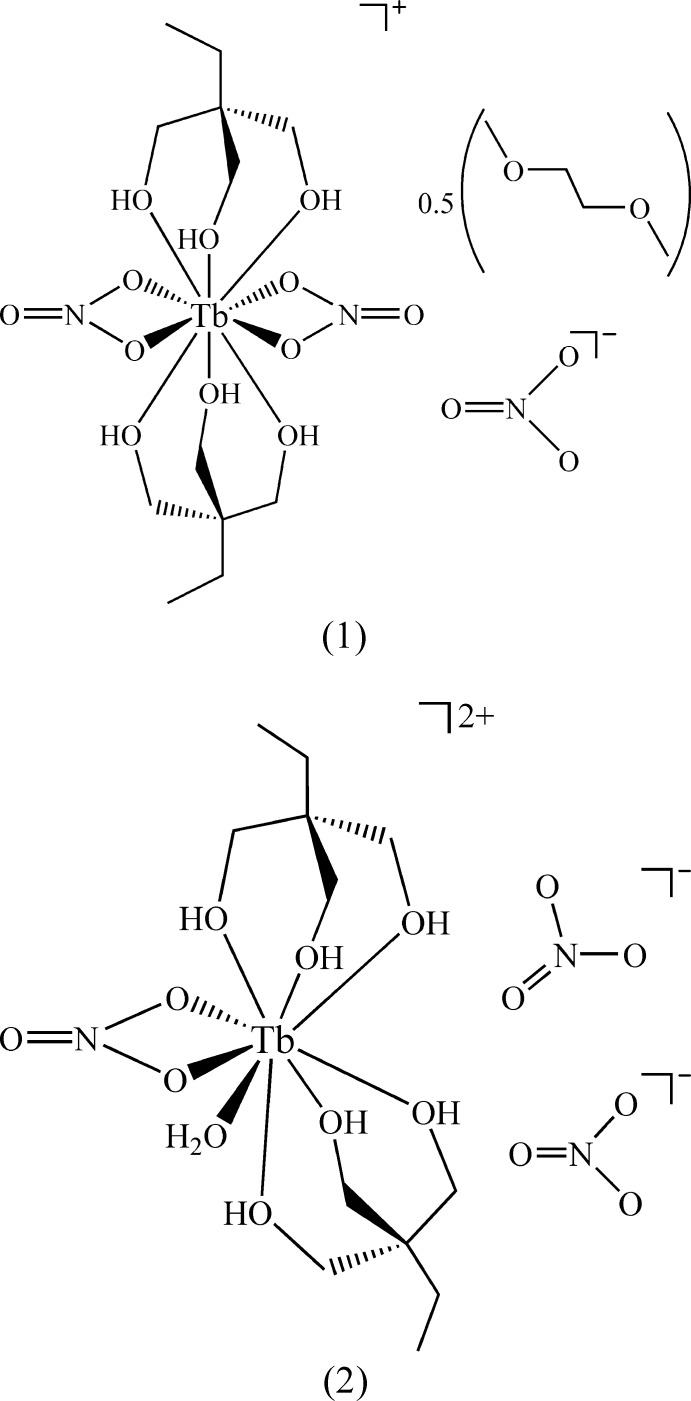


## Structural commentary   

**Figure 1:**
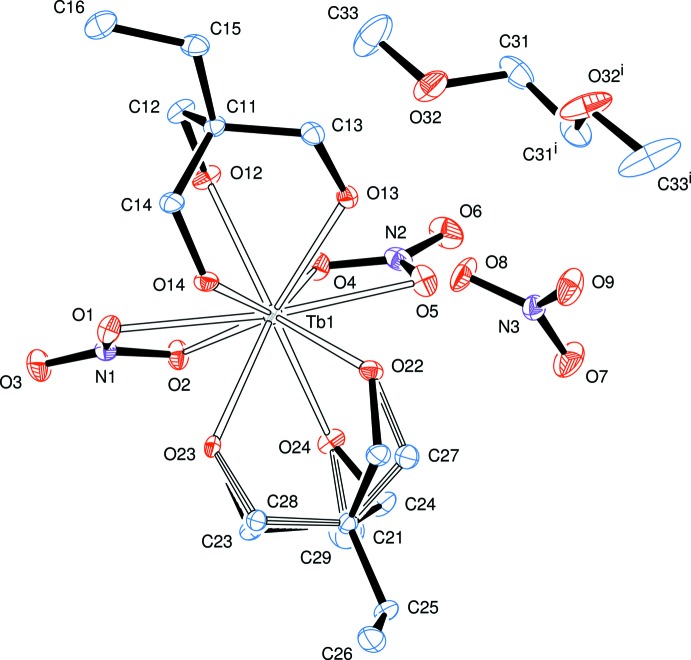
ORTEP representation of product **1**, [Tb(H_3_ *L* ^Et^)_2_(NO_3_)_2_](NO_3_)·0.5C_4_H_10_O_2_ (H_3_ *L* ^Et^ = 1,1,1-tris­(hy­droxy­meth­yl) propane and C_4_H_10_O_2_ = di­meth­oxy­ethane), with the atom-numbering scheme. There is disorder in the tripodal ligand of C21, with the minor component shown with striped bonds. Hydrogen atoms were omitted for clarity, and displacement ellipsoids are drawn at the 50% probability level. [Symmetry code: (i) −*x* + 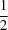, *y*, 1 − *z*.]

The crystals of product **1** contain the mononuclear complex [Tb(H_3_ *L* ^Et^)_2_(NO_3_)_2_](NO_3_)·0.5glyme (Fig. 1 ▸), in which the terbium(III) ion is 10-coordinate, being connected to six hydroxyl groups of the tripodal alcohol mol­ecules and to two bidentate nitrate ions. There is also one nitrate ion (acting as a counter-ion); a solvating di­meth­oxy­ethane (glyme) mol­ecule is shared between two units of the cationic complex. The complete gylme mol­ecule is completed by a crystallographic twofold rotation axis.

**Figure 2:**
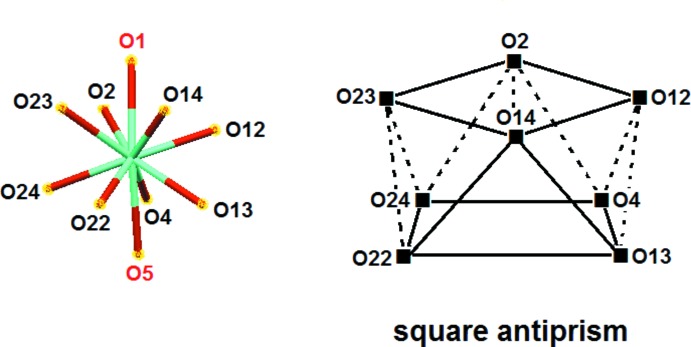
Plot of the coordination sphere (left) and schematic representation of the coordination environment about the terbium(III) atom in product **1**. The two mutually rotated square faces O2–O12–O14–O23 and O13—O22—O24—O4 are capped by atoms O1 and O5, respectively.

The geometric arrangement of the oxygen donor atoms about the metal atom in **1** is closer to a distorted *s*-bicapped square anti­prism, Fig. 2 ▸, than to an *s*-bicapped dodeca­hedron (Rohrbaugh & Jacobson, 1974 ▸). The choice of the bicapped square-anti­prismatic coordination sphere is mainly based on the angles between the coordinating oxygen atoms presented in Table 1 ▸, which are closer to the expected 90° values of the square planes in the former (Fig. 2 ▸) than to the alternating *ca* 77 and 100° angles in the latter (Rohrbaugh & Jacobson, 1974 ▸).

The mean square planes represented in Fig. 2 ▸ form a dihedral angle of 5.58° in the complex cation of **1**. The capping atoms, O1 and O5, both belong to the bidentate NO_3_^−^ ligands and form the two longest Tb—O bonds in the structure of **1**, 2.5697 (13) and 2.5874 (14) Å, respectively. Because of the typically small bite angles of the chelating nitrate ions, 49.50 (4)° for O2—Tb1—O1 and 50.12 (4)° for O4—Tb1—O5, the Tb—O1 and Tb—O5 bonds are significantly bent towards O2 and O4, respectively, creating additional structural distortion.

The average Tb—O bond involving the bidentate nitrate ligands in **1** [2.549 Å, and Table 2 ▸] is shorter than that described by Delangle and co-workers for the lanthanum(III) cation [La(H_3_ *L* ^1^)_2_(NO_3_)_2_]^+^, H_3_ *L* ^1^ = *cis*,*cis*-1,3,5-tri­hydroxy­cyclo­hexane; average = 2.681 Å; Delangle *et al.*, 2001 ▸]. This agrees with the smaller effective ionic radius of the Tb^III^ ion as compared to that of La^III^ (for example 1.095 *versus* 1.216 Å for nine-coordination respectively; Shannon, 1976 ▸). The effective ionic radius for 10-coordinate terbium(III) is not available in the literature. The mean Tb—O bond to the tripodal H_3_ *L* ^Et^ ligands is 2.404 Å, again significantly shorter than in the lanthanum(III)–cyclic triol analogue mentioned above (average = 2.542 Å). The lack of other reported lanthanide complexes with a bis­(tripodal alcohol)-bis(bidentate nitrate) coordination environment similar to that found in **1** restricts further comparisons.

The slow mixing of a hexane layer into the same reaction mixture that gave product **1** afforded another set of colourless crystals, product **2**, in high yield (see *Synthesis and crystallization*). As for **1**, crystals of **2** were practically insoluble at room temperature in hexane, toluene, thf, glyme and aceto­nitrile, but soluble in the last three solvents after heating at *ca* 323 K.

**Figure 3:**
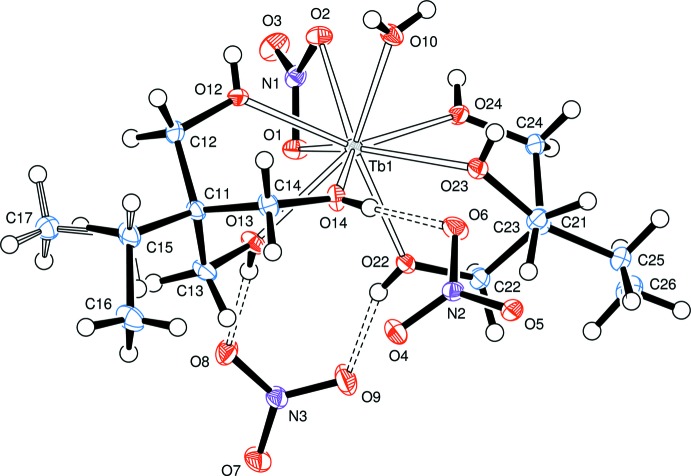
ORTEP representation of product **2**, [Tb(H_3_ *L* ^Et^)_2_(NO_3_)(H_2_O)](NO_3_)_2_, with the atom-numbering scheme. The terminal methyl group on C15 is disordered; the bonding of the minor component is shown with a striped bond. Displacement ellipsoids correspond to the 50% probability level. Hydrogen bonds are indicated by double-dashed lines.

Single-crystal X-ray diffraction analysis of **2** revealed again a mononuclear complex, this time of formula [Tb(H_3_ *L* ^Et^)_2_(NO_3_)(H_2_O)](NO_3_)_2_ (Fig. 3 ▸), in which the coordination number of the metal atom is nine. In this case, the terb­ium(III) atom is coordinated by six hydroxyl groups of the tripodal alcohols, a bidentate nitrate ion and one water mol­ecule probably coming from the Tb(NO_3_)_3_·5H_2_O starting material. Two distinct non-coordinating nitrate anions complete the charge balance in the product.

The geometry adopted by the metal atom in **2** is close to a tri-capped trigonal prism, as reported for complexes [*Ln*(H_3_ *L* ^1^)_2_(NO_3_)(H_2_O)](NO_3_)_2_ (*Ln* = Ho^III^, Eu^III^ and Yb^III^; H_3_ *L* ^1^ = *cis*,*cis*-1,3,5-tri­hydroxy­cyclo­hexane; Husson *et al.*, 1999 ▸; Delangle *et al.*, 2001 ▸). The two triangular faces, defined by O10–O12–O14 and O1–O22–O24, are nearly parallel, with a dihedral angle of 5.14° between the normals to the mean planes. The three rectangular faces, in turn, formed by O1–O12–O14–O22, O1–O10–O12–O24 and O10–O14–O22–O24, are capped by O13, O2 and O23, respectively. In these rectangular faces, the longer O⋯O distance is on average 3.345 Å, while the shorter is 2.961 Å (mean value). The alternative geometry of a monocapped square anti­prism, as described for [Y(H_3_ *L* ^Me^)_2_(NO_3_)(H_2_O)](NO_3_)_2_ (Chen *et al.*, 1997 ▸), appears less suitable to characterize **2** because of a much less regular placement of the coordinating oxygen atoms in the two square planes, O10–O12–O13–O23 and O1–O2–O22–O24, that are typical of this polyhedral arrangement.

The coordination of the Tb^III^ atom by the two tripodal ligands in both **1** and **2** is very similar. In the [*M*_3_ *M*′(*L* ^Et^)_2_(dpm)_3_] complexes (*M* and *M*′ = *d*-block metals), as above (Accorsi *et al.*, 2006 ▸; Totaro *et al.*, 2013 ▸; Westrup *et al.*, 2014 ▸; Gregoli *et al.*, 2009 ▸), the central metal is six-coordinate and the two tripodal ligands are inverted about that atom in an approximately octa­hedral arrangement; here, the C_B_⋯M ⋯C_B_′ angle is close to 180° (where C_B_ and C_B_′ are the bridgehead carbon atoms in the tripodal ligand). In our complexes **1** and **2**, with 10- and 9-coordinate atoms, the tripodal ligands are tilted apart, with C11—Tb1—C21 angles of 129.7 and 135.5°, respectively; this arrangement allows more space for the extra ligands in the coordination sphere. In both **1** and **2**, all the extra ligands, nitrate ions and water mol­ecules, lie on the plane that bis­ects the tripodal ligands; the number of extra coord­in­ating atoms determines the distribution in the bis­ecting plane and overall geometrical patterns, as described above.

According to Table 2 ▸, the metal–oxygen distances involving the H_3_ *L* ^Et^ ligands in **1** and **2** vary from 2.3583 (13) to 2.4749 (14) (complex **1**) and from 2.3545 (9) to 2.4344 (9) Å (complex **2**), these ranges being slightly larger than those reported for the *Ln* ^3+^ complexes of the tri­hydroxy­cyclo­hexane ligands (Delangle *et al.*, 2001 ▸). This probably arises from the different flexibilities of H_3_ *L* ^Et^ and the cyclic alcohols used in the syntheses, which allow for distortions of the lanthanide coordination environments. Also, the more crowded environment of the 10-coordinated metal ion in **1** as compared to **2** probably causes the larger observed variation.

The Tb—O bond lengths involving the nitrate ions in **2** are inter­mediate when compared to the analogous complexes of Eu^III^, Ho^III^ and Yb^III^ (Delangle *et al.*, 2001 ▸; Husson *et al.*, 1999 ▸) (Table 3 ▸). This is in agreement with the gradual decrease of the effective ionic radii of these ions (1.120, 1.095, 1.072 and 1.042 Å for Eu^III^, Tb^III^, Ho^III^ and Yb^III^, respectively, in 9-coordinate environments; Shannon, 1976 ▸). The same pattern is observed for the average metal–oxygen bond of the water mol­ecule (Table 3 ▸).

It has been demonstrated (Delangle *et al.*, 2001 ▸) that the formation of *Ln*(H_3_ *L*)_2_ complexes (*Ln* = La^III^, Pr^III^, Nd^III^, Eu^III^ and Yb^III^; *L* = *cis*,*cis*-1,3,5- or *cis*,*cis*-1,2,3-tri­hydroxy­cyclo­hexa­ne) in solution is strongly dependent on the metal:ligand ratio and on the chemical nature of the metal ion, its ionic radius, the polarity of the solvent and the nature of the counter-ion, either nitrate or triflate.

In the present work, the reaction between hydrated terbium(III) nitrate and H_3_ *L* ^Et^ led to the isolation of two distinct products, **1** and **2**, from the same reaction mixture, with modification only of the crystallization conditions. Product **2**, [Tb(H_3_ *L* ^Et^)_2_(NO_3_)(H_2_O)](NO_3_)_2_, was obtained in higher yield and after a shorter time inter­val (24 h) than the more symmetrical **1**, [Tb(H_3_ *L* ^Et^)_2_(NO_3_)_2_](NO_3_)·0.5glyme. The preparation of **2** is also easier to reproduce than that of **1**; the former appears to be favoured by addition of a less polar solvent (hexa­ne) to the reaction mixture. The isolation of **1**, on the other hand, seems to be subjected to a very subtle control of the crystallization conditions, and this is probably the reason why there are fewer reports of similar, anhydrous *Ln*(H_3_ *L*)_2_ products in the literature. The presence of solvating glyme in the crystals of **1** suggests that the use of other solvents with different stereo requirements could be a strategy to help the crystallization of this water-free complex.

## Supra­molecular features   

**Figure 4:**
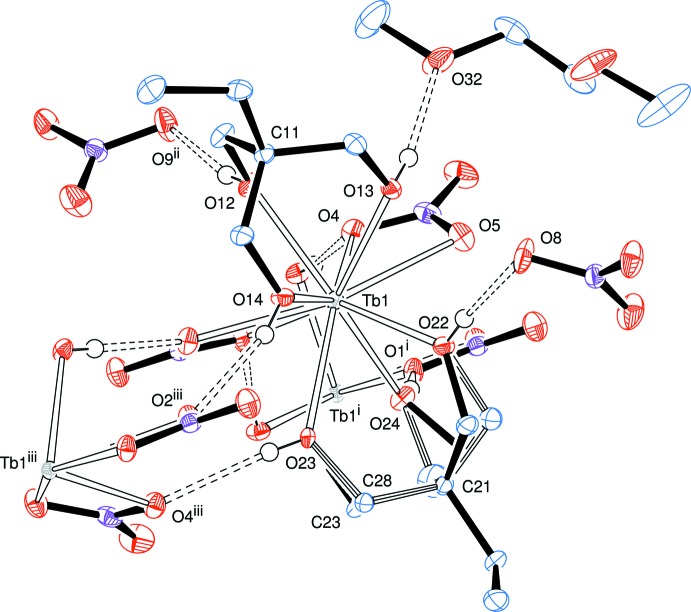
ORTEP representation of hydrogen bonding inter­actions about the ions and glyme mol­ecule of product **1**, Tb(H_3_ *L* ^Et^)_2_(NO_3_)_2_](NO_3_)·0.5C_4_H_10_O_2_, with hydrogen bonds indicated by double-dashed lines. Hydrogen atoms on carbon atoms have been omitted for clarity.

**Figure 5:**
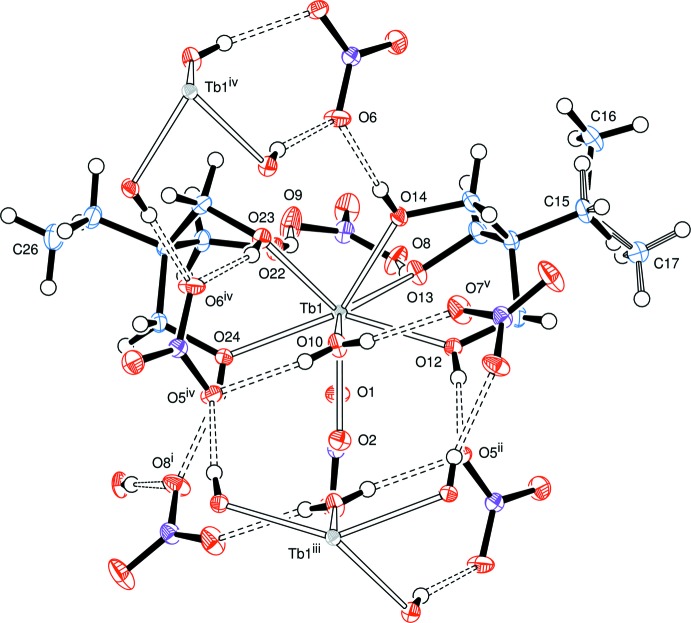
ORTEP representation of hydrogen bonding inter­actions about the ions of product **2**, [Tb(H_3_ *L* ^Et^)_2_(NO_3_)(H_2_O)](NO_3_)_2_, with hydrogen bonds indicated by double-dashed lines.

The three hydroxyl groups in both complexes are all donor groups to hydrogen bonds. The acceptor atoms are oxygen atoms of nitrate ions and, in complex **1**, an oxygen atom of the glyme mol­ecule (Fig. 4 ▸). In complex **2**, the water ligand forms two hydrogen bonds to two non-coordinating nitrate ions (Fig. 5 ▸). Thus, in both compounds, all the ions and the glyme mol­ecule are linked in an extensive three-dimensional hydrogen-bonded network.

In both complexes, there are also some inter­molecular C—H⋯O inter­actions, which may be described as ‘weak hydrogen bonds’. These are included in Tables 4 ▸ and 5 ▸ with the stronger O—H⋯O bonds.

## Database survey   

Delangle and co-workers (Delangle *et al.*, 2001 ▸; Husson *et al.*, 1999 ▸) reported the preparation of a variety of mononuclear complexes of various lanthanide(III) ions, specifically La^III^, Pr^III^, Nd^III^, Ho^III^, Eu^III^ and Yb^III^, with the trialcohols *cis*,*cis*-1,3,5-tri­hydroxy­cyclo­hexane (H_3_ *L* ^1^) and *cis*,*cis*-1,2,3-tri­hydroxy­cyclo­hexa­ne(H_3_ *L* ^2^) as models for the coordination of monosaccharides. In those compounds, as in **1** and **2**, the metal atoms are coordinated to two trialcohol mol­ecules and bidentate/monodentate *O-*donor anions (nitrate or triflate), or to these anions and water mol­ecules.

Monosaccharide-derived polyols have also been used as chelating ligands for lanthanide(III) ions. *Ln*Cl_3_ and *Ln*(NO_3_)_3_ (*Ln* = La^III^, Tb^III^ and Sm^III^) were shown to form chain-like complexes with d-galactitol in which the alditol provides three hydroxyl groups to coordinate one metal ion and three other hydroxyl groups to coordinate another; in all cases, there are two alditol mol­ecules bound to each lanthanide (Su *et al.*, 2002 ▸; Yu *et al.*, 2011 ▸). Other authors have employed erythritol, whose mol­ecule functions as two bidentate ligands or as a three-hydroxyl donor to a variety of lanthanide(III) chlorides (Ce, Pr, Nd, Eu, Gd and Tb; Yang *et al.*, 2012 ▸; Yang, Xie *et al.*, 2005 ▸; Yang, Xu *et al.*, 2005 ▸). These studies describe several possible binding modes of these polyols to lanthanide ions.

As far as tripodal alcohol ligands are concerned, mononuclear yttrium(III) complexes of 1,1,1-tris­(hy­droxy­meth­yl)propane (H_3_ *L* ^Et^) and 1,1,1-tris­(hy­droxy­meth­yl)ethane (H_3_ *L* ^Me^), as well as of the amino­polyalcohol (HOCH_2_)_3_CN(CH_2_CH_2_OH)_2_, H_5_ *L* ^N(EtOH)2^, were described by Chen and co-workers while investigating chelate complexes for radiotherapeutic applications (Chen *et al.*, 1997 ▸). In two of the reported products, those prepared from H_3_ *L* ^Me^ and H_5_ *L* ^N(EtOH)2^, the coordination sphere of the eight-coordinate yttrium atom contains chloride instead of nitrate ligands. A more recent study (Xu *et al.*, 2015 ▸), in its turn, describes a dysprosium(III) complex with H_3_ *L* ^Et^ that is isostructural to product **2** (present work) and has been employed to investigate possible biomedical applications of the binding of rare earth metal ions to the apoferritin protein.

## Synthesis and crystallization   

All experimental operations were performed under N_2(g)_ (99.999%, Praxair) or under vacuum of 10^−3^ Torr, using Schlenk and glove-box techniques. Solvents (di­meth­oxy­ethane and hexa­ne) were purified according to procedures described in the literature (Perrin & Armarego, 1997 ▸). Terbium(III) nitrate penta­hydrate and 1,1,1-tris­(hy­droxy­meth­yl)propane (H_3_ *L* ^Et^) were purchased from Aldrich; the latter was dissolved in thf/toluene (1:1), crystallized at 153 K, isolated by filtration and stored under N_2_ at room temperature prior to use. Elemental analysis (C, H and N) were performed under argon by MEDAC Laboratories Ltd. (Chobham, Surrey, UK), using a Thermal Scientific Flash EA 1112 Series Elemental Analyzer. Infrared spectra (FTIR, Nujol mulls) were obtained on a BIORAD FTS 3500GX instrument in the range of 400-4000 cm^−1^.

### Synthesis of [Tb(H_3_ *L* ^Et^)_2_(NO_3_)(H_2_O)](NO_3_)_2_·0.5glyme (product 1)   

A solution containing 1.91 g (4.39 mmol) of Tb(NO_3_)_3_·5H_2_O in 50 ml of di­meth­oxy­ethane (glyme) received the addition of 1.11 g (8.27 mmol) of solid 1,1,1-tris­(hy­droxy­meth­yl)propane to form a colourless solution that was refluxed for 15 min. After this period of time, the heating was turned off and a 32 ml aliquot of the reaction mixture was withdrawn for the isolation of product **2** (described below). The remaining 18 ml were cooled down to 153 K for four days, without forming any solid. The solution was then dried under vacuum and the resulting solid was almost completely redissolved in 7.5 ml of glyme. A fine suspension was obtained which, after seven days at 153 K, gave colourless crystals that were isolated and dried under vacuum (complex **1**). Yield: 360 mg, 0.547 mmol (12.5% based on the total amount of terbium employed in the reaction). If the yield was extrapolated to the total volume of the reaction mixture (50 ml) instead of the 18 ml effectively employed for crystallization, it could reach 34.7%. Elemental analysis: calculated for [Tb(H_3_ *L* ^Et^)_2_(NO_3_)_2_](NO_3_)·0.5glyme (C_14_H_33_N_3_O_16_Tb) C 25.54, H 5.05, N 6.38%. Found C 25.34, H 5.08, N 6.60%. FTIR (Nujol mull, cm^−1^, *s* = strong, *m* = medium, *w* = weak, *sh* = shoulder): 3359*m*, 3220*m* ν(O—H); 1050*sh*, 1020*s*, 942*s*, mainly ν(C—O); 1271*s* ν_a_(NO_2_), 1041*s* ν_s_(NO_2_).

### Isolation of [Tb(H_3_ *L* ^Et^)_2_(NO_3_)(H_2_O)](NO_3_)_2_ (product 2)   

The 32 ml aliquot of the reaction mixture described in the synthesis of **1** above received the careful addition of a hexane layer (20 ml) at room temperature, and was allowed to stand for 24 h. During this period it was possible to observe the formation of a large number of colourless crystals, which were isolated by filtration and dried under vacuum (complex **2**). Yield: 1.36 g, 2.15 mmol (49.1% based on the total amount of terbium employed in the reaction). If the yield was extrap­olated to the total volume of the reaction mixture (50 ml) instead of 32 ml actually employed for crystallization of **2**, this yield could reach 76.7%). Elemental analysis: calculated for C_12_H_30_N_3_O_16_Tb C 22.83, H 4.79, N 6.66%. Found C 22.69, H 4.84, N 6.78. FTIR (Nujol mull, cm^−1^): 3475*m*, 3350*s*, 3184*s* ν(O—H); 1620*w* δ(O—H), 1050*s*, 1035*s*, 949*s*, mainly ν(C—O); 1278*s* ν_a_(NO_2_), 1037*s* ν_s_(NO_2_).

## Refinement   

Crystal data, data collection and structure refinement details for both complexes **1** and **2** are summarized in Table 6 ▸.

Disorder was noted in both structures: in compound **1**, the methyl­ene groups in the three CH_2_OH groups in one tripodal ligand were each found to be disordered over two sets of sites, with an occupancy ratio of 0.911 (7) : 0.089 (7), whereas in **2**, the disorder is in a terminal methyl group, which is disordered over two orientations, with an occupancy ratio of 0.827 (4) : 0.173 (4).

All the hydroxyl and water hydrogen atoms were located clearly in difference maps and were refined freely and satisfactorily. All the remaining hydrogen atoms were set in idealized positions and refined as riding on the parent carbon atoms.

## Supplementary Material

Crystal structure: contains datablock(s) Compound-1, Compound-2, global. DOI: 10.1107/S2056989017001116/hb7653sup1.cif

Structure factors: contains datablock(s) Compound-1. DOI: 10.1107/S2056989017001116/hb7653Compound-1sup2.hkl

Supporting information file. DOI: 10.1107/S2056989017001116/hb7653Compound-1sup4.cdx

Structure factors: contains datablock(s) Compound-2. DOI: 10.1107/S2056989017001116/hb7653Compound-2sup3.hkl

Supporting information file. DOI: 10.1107/S2056989017001116/hb7653Compound-2sup5.cdx

CCDC references: 1529079, 1529078

Additional supporting information:  crystallographic information; 3D view; checkCIF report

## Figures and Tables

**Table 1:** Selected non-bonding angles (°) in the mol­ecular structure of product **1**

O24⋯O22⋯O13	101.47	O2⋯O23⋯O14	86.82
O22⋯O13⋯O4	83.25	O23⋯O14⋯O12	100.40
O13⋯O4⋯O24	81.56	O14⋯O12⋯O2	86.34
O4⋯O24⋯O22	93.06	O12⋯O2⋯O23	84.98

**Table 2:** Metal–oxygen distances (Å) in the two complexes, **1** and **2**

Complex **1**		Complex **2**	
Tb1—O1	2.5697 (13)	Tb1—O1	2.4706 (10)
Tb1—O2	2.5418 (13)	Tb1—O2	2.4762 (9)
Tb1—O4	2.4953 (13)	Tb1—O10	2.3786 (9)
Tb1—O5	2.5874 (14)		
Tb1—O12	2.4078 (13)	Tb1—O12	2.3597 (9)
Tb1—O13	2.4245 (14)	Tb1—O13	2.4119 (9)
Tb1—O14	2.3810 (14)	Tb1—O14	2.3545 (9)
Tb1—O22	2.3583 (13)	Tb1—O22	2.3734 (9)
Tb1—O23	2.4749 (14)	Tb1—O23	2.4344 (9)
Tb1—O24	2.3790 (13)	Tb1—O24	2.4112 (9)
			
O12—Tb1—O13	66.85 (5)	O12—Tb1—O13	68.99 (3)
O14—Tb1—O12	76.53 (5)	O14—Tb1—O12	72.38 (3)
O22—Tb1—O12	136.11 (5)	O12—Tb1—O22	140.67 (3)
O12—Tb1—O23	130.08 (5)	O12—Tb1—O23	131.17 (3)
O24—Tb1—O12	147.08 (5)	O12—Tb1—O24	141.14 (3)
O14—Tb1—O13	70.11 (5)	O14—Tb1—O13	70.72 (3)
O22—Tb1—O13	71.20 (5)	O22—Tb1—O13	71.69 (3)
O13—Tb1—O23	129.41 (5)	O13—Tb1—O23	123.37 (3)
O24—Tb1—O13	128.37 (5)	O24—Tb1—O13	134.33 (3)
O22—Tb1—O14	77.75 (5)	O14—Tb1—O22	93.64 (3)
O14—Tb1—O23	70.24 (5)	O14—Tb1—O23	69.97 (3)
O24—Tb1—O14	133.94 (5)	O14—Tb1—O24	138.28 (3)
O22—Tb1—O23	70.76 (5)	O22—Tb1—O23	71.90 (3)
O22—Tb1—O24	72.47 (5)	O22—Tb1—O24	72.06 (3)
O24—Tb1—O23	67.19 (5)	O24—Tb1—O23	68.34 (3)
			
O2—Tb1—O1	49.50 (4)	O1—Tb1—O2	51.89 (3)
O4—Tb1—O1	104.45 (4)	O10—Tb1—O1	124.95 (3)
O1—Tb1—O5	154.57 (4)	O10—Tb1—O2	73.09 (3)
O4—Tb1—O2	62.06 (4)		
O2—Tb1—O5	107.78 (4)		
O4—Tb1—O5	50.12 (4)		
O12—Tb1—O1	69.27 (5)	O12—Tb1—O1	88.98 (3)
O13—Tb1—O1	125.17 (4)	O13—Tb1—O1	71.02 (3)
O14—Tb1—O1	69.06 (4)	O14—Tb1—O1	141.34 (3)
O22—Tb1—O1	130.92 (4)	O22—Tb1—O1	79.51 (3)
O23—Tb1—O1	64.60 (4)	O23—Tb1—O1	139.42 (3)
O24—Tb1—O1	106.31 (4)	O24—Tb1—O1	75.88 (3)
O12—Tb1—O2	80.97 (5)	O12—Tb1—O2	73.16 (3)
O13—Tb1—O2	144.10 (5)	O13—Tb1—O2	110.27 (3)
O14—Tb1—O2	118.55 (4)	O14—Tb1—O2	142.17 (3)
O22—Tb1—O2	142.88 (5)	O22—Tb1—O2	123.21 (3)
O23—Tb1—O2	83.39 (4)	O23—Tb1—O2	125.81 (3)
O24—Tb1—O2	73.14 (4)	O24—Tb1—O2	69.33 (3)
O12—Tb1—O4	72.71 (5)	O12—Tb1—O10	75.77 (3)
O13—Tb1—O4	92.32 (5)	O10—Tb1—O13	141.19 (3)
O14—Tb1—O4	148.71 (4)	O14—Tb1—O10	83.74 (3)
O22—Tb1—O4	122.01 (4)	O22—Tb1—O10	140.58 (3)
O23—Tb1—O4	136.38 (5)	O10—Tb1—O23	70.30 (3)
O24—Tb1—O4	77.30 (5)	O10—Tb1—O24	83.96 (3)
O12—Tb1—O5	98.74 (5)		
O13—Tb1—O5	64.19 (5)		
O14—Tb1—O5	131.61 (5)		
O22—Tb1—O5	73.45 (4)		
O23—Tb1—O5	131.18 (4)		
O24—Tb1—O5	71.11 (5)		

**Table 3:** Bond lengths (Å) involving the metal cations and the nitrate/water ligands in the lanthanide complexes [Tb(H_3_ *L*Et)_2_(NO_3_)(H_2_O)](NO_3_)_2_ *^*a*^* and [*Ln*(H_3_ *L* ^1^)(NO_3_)(H_2_O)](NO_3_)_2_ (*Ln* = Eu^III^, Ho^III^ and Yb^III^; H_3_ *L* ^1^ = *cis*,*cis*-1,3,5-tri­hydroxy­cyclo­hexa­ne)^*b*^

Eu—O(NO_3_)	2.4869 (12)	Eu—O(NO_3_)	2.517 (2)	Eu—O(H_2_O)	2.4279 (14)
Tb—O(NO_3_)	2.4706 (10)	Tb—O(NO_3_)	2.4762 (9)	Tb—O(H_2_O)	2.3786 (9)
Ho—O(NO_3_)	2.450 (9)	Ho—O(NO_3_)	2.454 (8)	Ho—O(H_2_O)	2.377 (8)
Yb—O(NO_3_)	2.448 (6)	Yb—O(NO_3_)	2.439 (7)	Yb—O(H_2_O)	2.331 (7)

Notes: (*a*) this work, product **2**; (*b*) Delangle *et al.* (2001); Husson *et al.* (1999).

**Table 4:** Hydrogen-bond geometry (Å, °) for **1** [Chem scheme1]

*D*—H⋯*A*	*D*—H	H⋯*A*	*D*⋯*A*	*D*—H⋯*A*
O12—H12*O*⋯O9^i^	0.72 (2)	1.98 (2)	2.683 (2)	167 (3)
O13—H13*O*⋯O32	0.74 (2)	2.04 (2)	2.774 (2)	170 (3)
O14—H14*O*⋯O2^ii^	0.74 (2)	2.07 (3)	2.7935 (19)	165 (3)
O22—H22*O*⋯O8	0.73 (2)	2.01 (3)	2.735 (2)	172 (3)
O23—H23*O*⋯O4^ii^	0.69 (2)	2.16 (2)	2.8550 (19)	175 (2)
O24—H24*O*⋯O1^iii^	0.74 (3)	1.93 (3)	2.6624 (19)	171 (3)
C22—H22*B*⋯O3^iv^	0.99	2.44	3.358 (3)	153
C24—H24*B*⋯O7^v^	0.99	2.49	3.220 (3)	130
C29—H29*A*⋯O7^v^	0.99	2.41	3.27 (3)	146

Symmetry codes: (i) 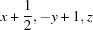; (ii) 
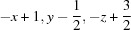
; (iii) 
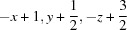
; (iv) 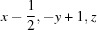; (v) 
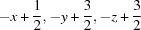
.

**Table 5:** Hydrogen-bond geometry (Å, °) for **2** [Chem scheme1]

*D*—H⋯*A*	*D*—H	H⋯*A*	*D*⋯*A*	*D*—H⋯*A*
O10—H1*OA*⋯O7^i^	0.75 (2)	2.03 (2)	2.7420 (14)	159 (2)
O10—H1*OB*⋯O5^ii^	0.79 (2)	2.00 (2)	2.7703 (14)	167 (2)
O13—H13*O*⋯O8	0.77 (2)	1.91 (2)	2.6695 (14)	169 (2)
O12—H12*O*⋯O5^iii^	0.74 (2)	1.93 (2)	2.6713 (13)	174 (2)
O14—H14*O*⋯O6	0.73 (2)	1.97 (2)	2.6992 (14)	174 (2)
O23—H23*O*⋯O6^ii^	0.71 (2)	2.09 (2)	2.7669 (14)	161 (2)
O22—H22*O*⋯O9	0.76 (2)	1.94 (2)	2.6609 (14)	157 (2)
O24—H24*O*⋯O8^iv^	0.73 (2)	1.97 (2)	2.6650 (14)	158 (2)
C14—H14*A*⋯O7^i^	0.99	2.58	3.3462 (17)	135
C23—H23*A*⋯O3^v^	0.99	2.51	3.4003 (16)	149

Symmetry codes: (i) 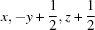; (ii) 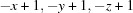; (iii) 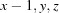; (iv) 
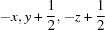
; (v) 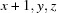.

**Table 6:** Experimental details

	**1**	**2**
Crystal data		
Chemical formula	[Tb(NO_3_)_2_(C_6_H_14_O_3_)_2_]NO_3_·0.5C_4_H_10_O_2_	[Tb(NO_3_)(C_6_H_14_O_3_)_2_(H_2_O)](NO_3_)_2_
*M*_r_	658.35	631.31
Crystal system, space group	Monoclinic, *I*2/*a*	Monoclinic, *P*2_1_/*c*
Temperature (K)	100	100
*a*, *b*, *c* (Å)	20.1864 (13), 10.2997 (6), 23.832 (2)	9.1440 (6), 12.7870 (7), 19.7151 (12)
β (°)	108.004 (3)	101.796 (2)
*V* (Å^3^)	4712.4 (6)	2256.5 (2)
*Z*	8	4
Radiation type	Mo *K*α	Mo *K*α
μ (mm^−1^)	3.08	3.22
Crystal size (mm)	0.14 × 0.11 × 0.09	0.36 × 0.19 × 0.18

Data collection		
Diffractometer	Bruker D8 VENTURE/PHOTON100 CMOS	Bruker D8 VENTURE/PHOTON100 CMOS
Absorption correction	Multi-scan (*SADABS*; Bruker, 2014 ▸)	Multi-scan (*SADABS*; Bruker, 2014 ▸)
*T*_min_, *T*_max_	0.694, 0.746	0.636, 0.746
No. of measured, independent and observed [*I* > 2σ(*I*)] reflections	172878, 5889, 5031	244328, 5629, 5542
*R*_int_	0.071	0.023
(sin θ/λ)_max_ (Å^−1^)	0.670	0.670

Refinement		
*R*[*F* ^2^ > 2σ(*F* ^2^)], *wR*(*F* ^2^), *S*	0.018, 0.036, 1.07	0.012, 0.029, 1.13
No. of reflections	5889	5629
No. of parameters	344	326
H-atom treatment	H atoms treated by a mixture of independent and constrained refinement	H atoms treated by a mixture of independent and constrained refinement
Δρ_max_, Δρ_min_ (e Å^−3^)	0.77, −0.54	0.74, −0.26

Computer programs: *APEX3* (Bruker, 2015 ▸), *SAINT* (Bruker, 2013 ▸), *SHELXS97* (Sheldrick 2008 ▸), *SHELXL2014* (Sheldrick, 2015 ▸), *ORTEP* (Johnson, 1976 ▸), *ORTEP-3* and *WinGX* (Farrugia, 2012 ▸).

## supplementary crystallographic information

### (Compound-1) Dinitratobis[1,1,1-tris(hydroxymethyl)propane]terbium(III) nitrate dimethoxyethane hemisolvate Crystal data

**Table:**

[Tb(NO_3_)_2_(C_6_H_14_O_3_)_2_]NO_3_·0.5C_4_H_10_O_2_	*F*(000) = 2648
*M**_r_* = 658.35	*D*_x_ = 1.856 Mg m^−^^3^
Monoclinic, *I*2/*a*	Mo *K*α radiation, λ = 0.71073 Å
*a* = 20.1864 (13) Å	Cell parameters from 9404 reflections
*b* = 10.2997 (6) Å	θ = 3.1–28.2°
*c* = 23.832 (2) Å	µ = 3.08 mm^−^^1^
β = 108.004 (3)°	*T* = 100 K
*V* = 4712.4 (6) Å^3^	Fragment, colourless
*Z* = 8	0.14 × 0.11 × 0.09 mm

### (Compound-1) Dinitratobis[1,1,1-tris(hydroxymethyl)propane]terbium(III) nitrate dimethoxyethane hemisolvate Data collection

**Table:**

Bruker D8 VENTURE/PHOTON100 CMOS diffractometer	5889 independent reflections
Radiation source: fine-focus sealed tube	5031 reflections with *I* > 2σ(*I*)
Graphite monochromator	*R*_int_ = 0.071
Detector resolution: 10.4167 pixels mm^-1^	θ_max_ = 28.4°, θ_min_ = 2.7°
φ and ω scans	*h* = −26→26
Absorption correction: multi-scan (SADABS; Bruker, 2014)	*k* = −13→13
*T*_min_ = 0.694, *T*_max_ = 0.746	*l* = −31→31
172878 measured reflections	

### (Compound-1) Dinitratobis[1,1,1-tris(hydroxymethyl)propane]terbium(III) nitrate dimethoxyethane hemisolvate Refinement

**Table:**

Refinement on *F*^2^	Primary atom site location: structure-invariant direct methods
Least-squares matrix: full	Secondary atom site location: difference Fourier map
*R*[*F*^2^ > 2σ(*F*^2^)] = 0.018	Hydrogen site location: mixed
*wR*(*F*^2^) = 0.036	H atoms treated by a mixture of independent and constrained refinement
*S* = 1.07	*w* = 1/[σ^2^(*F*_o_^2^) + (0.015*P*)^2^ + 5.6056*P*] where *P* = (*F*_o_^2^ + 2*F*_c_^2^)/3
5889 reflections	(Δ/σ)_max_ = 0.001
344 parameters	Δρ_max_ = 0.77 e Å^−^^3^
0 restraints	Δρ_min_ = −0.54 e Å^−^^3^

### (Compound-1) Dinitratobis[1,1,1-tris(hydroxymethyl)propane]terbium(III) nitrate dimethoxyethane hemisolvate Special details

**Table:**

Geometry. All esds (except the esd in the dihedral angle between two l.s. planes) are estimated using the full covariance matrix. The cell esds are taken into account individually in the estimation of esds in distances, angles and torsion angles; correlations between esds in cell parameters are only used when they are defined by crystal symmetry. An approximate (isotropic) treatment of cell esds is used for estimating esds involving l.s. planes.

### (Compound-1) Dinitratobis[1,1,1-tris(hydroxymethyl)propane]terbium(III) nitrate dimethoxyethane hemisolvate Fractional atomic coordinates and isotropic or equivalent isotropic displacement parameters (Å^2^)

**Table:**

	*x*	*y*	*z*	*U*_iso_*/*U*_eq_	Occ. (<1)
Tb1	0.41961 (2)	0.52548 (2)	0.68945 (2)	0.00881 (3)	
C11	0.39871 (10)	0.29809 (17)	0.56884 (8)	0.0118 (4)	
C12	0.46190 (10)	0.38204 (19)	0.57107 (9)	0.0160 (4)	
H12A	0.5040	0.3266	0.5811	0.019*	
H12B	0.4555	0.4198	0.5315	0.019*	
O12	0.47253 (7)	0.48486 (14)	0.61340 (6)	0.0149 (3)	
C13	0.33152 (10)	0.37790 (18)	0.55500 (8)	0.0140 (4)	
H13A	0.3195	0.4096	0.5139	0.017*	
H13B	0.2933	0.3203	0.5576	0.017*	
O13	0.33531 (7)	0.48753 (14)	0.59343 (6)	0.0131 (3)	
C14	0.41005 (10)	0.21802 (18)	0.62473 (8)	0.0136 (4)	
H14A	0.3753	0.1471	0.6172	0.016*	
H14B	0.4570	0.1784	0.6361	0.016*	
O14	0.40358 (7)	0.29827 (13)	0.67214 (6)	0.0129 (3)	
C15	0.38567 (10)	0.20335 (19)	0.51616 (8)	0.0169 (4)	
H15A	0.3415	0.1567	0.5119	0.020*	
H15B	0.3790	0.2553	0.4799	0.020*	
C16	0.44210 (11)	0.1029 (2)	0.51961 (10)	0.0239 (5)	
H16A	0.4283	0.0486	0.4841	0.036*	
H16B	0.4860	0.1471	0.5224	0.036*	
H16C	0.4483	0.0483	0.5545	0.036*	
C21	0.34085 (9)	0.52402 (18)	0.80793 (8)	0.0116 (3)	
C22	0.29008 (11)	0.4608 (3)	0.75328 (9)	0.0142 (6)	0.911 (7)
H22A	0.2903	0.3655	0.7588	0.017*	0.911 (7)
H22B	0.2423	0.4928	0.7481	0.017*	0.911 (7)
C23	0.40750 (13)	0.4457 (3)	0.83121 (10)	0.0131 (6)	0.911 (7)
H23A	0.4426	0.4973	0.8611	0.016*	0.911 (7)
H23B	0.3980	0.3658	0.8505	0.016*	0.911 (7)
C24	0.35697 (14)	0.6637 (2)	0.79430 (12)	0.0152 (6)	0.911 (7)
H24A	0.3141	0.7049	0.7684	0.018*	0.911 (7)
H24B	0.3727	0.7140	0.8315	0.018*	0.911 (7)
O22	0.30941 (7)	0.49039 (13)	0.70161 (6)	0.0129 (3)	
O23	0.43465 (7)	0.41116 (14)	0.78392 (6)	0.0128 (3)	
O24	0.41020 (7)	0.66656 (14)	0.76566 (6)	0.0140 (3)	
C25	0.30551 (10)	0.5344 (2)	0.85686 (8)	0.0165 (4)	
H25A	0.3404	0.5677	0.8930	0.020*	
H25B	0.2679	0.5999	0.8444	0.020*	
C26	0.27454 (10)	0.4102 (2)	0.87266 (9)	0.0207 (4)	
H26A	0.2537	0.4284	0.9039	0.031*	
H26B	0.2386	0.3773	0.8377	0.031*	
H26C	0.3113	0.3450	0.8865	0.031*	
C27	0.2836 (12)	0.521 (3)	0.7509 (10)	0.015 (6)*	0.089 (7)
H27A	0.2488	0.4550	0.7530	0.018*	0.089 (7)
H27B	0.2601	0.6065	0.7439	0.018*	0.089 (7)
C28	0.3854 (14)	0.399 (3)	0.8212 (11)	0.011 (6)*	0.089 (7)
H28A	0.4119	0.3927	0.8636	0.013*	0.089 (7)
H28B	0.3556	0.3206	0.8096	0.013*	0.089 (7)
C29	0.387 (2)	0.647 (3)	0.8196 (16)	0.032 (8)*	0.089 (7)
H29A	0.3594	0.7234	0.8254	0.039*	0.089 (7)
H29B	0.4273	0.6362	0.8553	0.039*	0.089 (7)
N2	0.39273 (9)	0.79057 (15)	0.63712 (7)	0.0162 (3)	
O4	0.45380 (7)	0.74010 (12)	0.65838 (6)	0.0164 (3)	
O5	0.34311 (7)	0.72241 (13)	0.64212 (6)	0.0190 (3)	
O6	0.38490 (8)	0.89771 (13)	0.61430 (6)	0.0255 (3)	
N1	0.57343 (8)	0.50126 (15)	0.75057 (7)	0.0130 (3)	
O1	0.53526 (7)	0.40279 (12)	0.73137 (6)	0.0148 (3)	
O2	0.54149 (7)	0.60919 (12)	0.74203 (6)	0.0162 (3)	
O3	0.63542 (7)	0.49260 (14)	0.77547 (6)	0.0230 (3)	
N3	0.13781 (8)	0.45279 (15)	0.62056 (7)	0.0161 (3)	
O7	0.13714 (8)	0.54895 (15)	0.65113 (7)	0.0290 (4)	
O8	0.19346 (7)	0.41340 (15)	0.61347 (7)	0.0259 (3)	
O9	0.08179 (7)	0.39286 (14)	0.59578 (7)	0.0259 (3)	
C31	0.27954 (11)	0.7871 (2)	0.48781 (10)	0.0258 (5)	
H31A	0.2616	0.7785	0.4443	0.031*	
H31B	0.3048	0.8707	0.4972	0.031*	
O32	0.32611 (10)	0.68347 (15)	0.51152 (7)	0.0355 (4)	
C33	0.38661 (16)	0.6912 (3)	0.49237 (13)	0.0530 (9)	
H33A	0.4180	0.6188	0.5092	0.079*	
H33B	0.3726	0.6864	0.4492	0.079*	
H33C	0.4107	0.7735	0.5055	0.079*	
H12O	0.5042 (13)	0.518 (2)	0.6139 (11)	0.019 (6)*	
H13O	0.3300 (12)	0.544 (2)	0.5736 (11)	0.021 (7)*	
H14O	0.4146 (12)	0.255 (2)	0.6983 (11)	0.026 (7)*	
H22O	0.2797 (13)	0.473 (2)	0.6760 (11)	0.022 (7)*	
H23O	0.4631 (12)	0.371 (2)	0.7967 (10)	0.013 (6)*	
H24O	0.4214 (13)	0.735 (3)	0.7651 (11)	0.030 (8)*	

### (Compound-1) Dinitratobis[1,1,1-tris(hydroxymethyl)propane]terbium(III) nitrate dimethoxyethane hemisolvate Atomic displacement parameters (Å^2^)

**Table:**

	*U*^11^	*U*^22^	*U*^33^	*U*^12^	*U*^13^	*U*^23^
Tb1	0.00815 (4)	0.00833 (4)	0.01016 (4)	−0.00074 (4)	0.00313 (3)	−0.00065 (4)
C11	0.0138 (9)	0.0102 (9)	0.0119 (9)	−0.0002 (7)	0.0047 (7)	−0.0012 (7)
C12	0.0169 (10)	0.0159 (10)	0.0182 (10)	−0.0023 (8)	0.0101 (8)	−0.0046 (8)
O12	0.0120 (7)	0.0175 (7)	0.0177 (7)	−0.0066 (6)	0.0084 (6)	−0.0060 (6)
C13	0.0147 (9)	0.0129 (9)	0.0124 (9)	−0.0012 (7)	0.0011 (7)	−0.0019 (7)
O13	0.0141 (7)	0.0121 (7)	0.0125 (7)	0.0023 (5)	0.0032 (5)	0.0014 (6)
C14	0.0192 (10)	0.0099 (9)	0.0114 (9)	0.0007 (7)	0.0041 (8)	−0.0029 (7)
O14	0.0190 (7)	0.0103 (7)	0.0103 (7)	0.0005 (5)	0.0057 (6)	0.0005 (5)
C15	0.0221 (10)	0.0155 (10)	0.0124 (9)	−0.0016 (8)	0.0042 (8)	−0.0032 (7)
C16	0.0303 (12)	0.0196 (11)	0.0241 (11)	0.0009 (9)	0.0118 (10)	−0.0079 (9)
C21	0.0110 (8)	0.0124 (8)	0.0128 (9)	0.0010 (7)	0.0055 (7)	0.0006 (8)
C22	0.0124 (10)	0.0181 (16)	0.0134 (11)	−0.0013 (9)	0.0061 (8)	0.0014 (9)
C23	0.0127 (11)	0.0166 (14)	0.0120 (11)	0.0006 (10)	0.0068 (9)	0.0002 (9)
C24	0.0184 (13)	0.0124 (11)	0.0193 (14)	0.0000 (9)	0.0123 (11)	−0.0014 (9)
O22	0.0095 (6)	0.0187 (7)	0.0100 (6)	−0.0025 (5)	0.0023 (5)	−0.0001 (5)
O23	0.0121 (7)	0.0145 (7)	0.0130 (7)	0.0068 (6)	0.0054 (6)	0.0023 (6)
O24	0.0177 (7)	0.0089 (7)	0.0183 (7)	−0.0036 (6)	0.0097 (6)	−0.0017 (5)
C25	0.0159 (9)	0.0225 (10)	0.0133 (9)	0.0032 (8)	0.0078 (7)	−0.0008 (8)
C26	0.0161 (10)	0.0303 (12)	0.0174 (10)	0.0005 (9)	0.0073 (8)	0.0066 (9)
N2	0.0232 (9)	0.0114 (8)	0.0118 (8)	−0.0009 (7)	0.0022 (7)	−0.0012 (6)
O4	0.0175 (7)	0.0123 (7)	0.0202 (7)	−0.0008 (5)	0.0070 (6)	0.0003 (5)
O5	0.0174 (7)	0.0143 (7)	0.0224 (7)	−0.0022 (6)	0.0019 (6)	0.0012 (6)
O6	0.0418 (10)	0.0099 (7)	0.0229 (8)	0.0022 (6)	0.0071 (7)	0.0055 (6)
N1	0.0133 (8)	0.0153 (9)	0.0110 (7)	0.0010 (6)	0.0047 (6)	0.0014 (6)
O1	0.0137 (7)	0.0100 (7)	0.0217 (7)	−0.0011 (5)	0.0070 (6)	−0.0012 (5)
O2	0.0142 (7)	0.0118 (7)	0.0208 (7)	0.0016 (5)	0.0025 (6)	−0.0004 (5)
O3	0.0102 (7)	0.0329 (9)	0.0228 (8)	0.0021 (6)	0.0005 (6)	0.0012 (6)
N3	0.0134 (8)	0.0182 (9)	0.0159 (8)	0.0013 (6)	0.0034 (7)	−0.0021 (6)
O7	0.0255 (8)	0.0269 (9)	0.0339 (9)	0.0024 (6)	0.0080 (7)	−0.0160 (7)
O8	0.0113 (7)	0.0347 (9)	0.0321 (9)	0.0014 (6)	0.0073 (6)	−0.0159 (7)
O9	0.0124 (7)	0.0275 (8)	0.0363 (9)	−0.0021 (6)	0.0053 (7)	−0.0127 (7)
C31	0.0334 (13)	0.0159 (10)	0.0204 (11)	−0.0028 (9)	−0.0029 (9)	0.0044 (8)
O32	0.0599 (12)	0.0252 (9)	0.0351 (9)	0.0212 (8)	0.0351 (9)	0.0164 (7)
C33	0.080 (2)	0.0396 (16)	0.065 (2)	0.0328 (15)	0.0601 (18)	0.0299 (14)

### (Compound-1) Dinitratobis[1,1,1-tris(hydroxymethyl)propane]terbium(III) nitrate dimethoxyethane hemisolvate Geometric parameters (Å, º)

**Table:**

Tb1—O22	2.3583 (13)	C23—O23	1.442 (2)
Tb1—O24	2.3790 (13)	C23—H23A	0.9900
Tb1—O14	2.3810 (13)	C23—H23B	0.9900
Tb1—O12	2.4078 (13)	C24—O24	1.440 (2)
Tb1—O13	2.4245 (13)	C24—H24A	0.9900
Tb1—O23	2.4749 (14)	C24—H24B	0.9900
Tb1—O4	2.4953 (13)	O22—C27	1.46 (2)
Tb1—O2	2.5418 (13)	O22—H22O	0.73 (2)
Tb1—O1	2.5697 (13)	O23—C28	1.53 (2)
Tb1—O5	2.5874 (14)	O23—H23O	0.69 (2)
C11—C14	1.523 (2)	O24—C29	1.52 (3)
C11—C12	1.528 (3)	O24—H24O	0.74 (3)
C11—C13	1.532 (3)	C25—C26	1.521 (3)
C11—C15	1.547 (3)	C25—H25A	0.9900
C12—O12	1.432 (2)	C25—H25B	0.9900
C12—H12A	0.9900	C26—H26A	0.9800
C12—H12B	0.9900	C26—H26B	0.9800
O12—H12O	0.72 (2)	C26—H26C	0.9800
C13—O13	1.441 (2)	C27—H27A	0.9900
C13—H13A	0.9900	C27—H27B	0.9900
C13—H13B	0.9900	C28—H28A	0.9900
O13—H13O	0.74 (2)	C28—H28B	0.9900
C14—O14	1.438 (2)	C29—H29A	0.9900
C14—H14A	0.9900	C29—H29B	0.9900
C14—H14B	0.9900	N2—O6	1.219 (2)
O14—H14O	0.74 (2)	N2—O5	1.259 (2)
C15—C16	1.522 (3)	N2—O4	1.289 (2)
C15—H15A	0.9900	N1—O3	1.211 (2)
C15—H15B	0.9900	N1—O2	1.2696 (19)
C16—H16A	0.9800	N1—O1	1.270 (2)
C16—H16B	0.9800	N3—O7	1.232 (2)
C16—H16C	0.9800	N3—O8	1.254 (2)
C21—C27	1.49 (2)	N3—O9	1.263 (2)
C21—C23	1.519 (3)	C31—O32	1.419 (3)
C21—C24	1.532 (3)	C31—C31^i^	1.479 (5)
C21—C22	1.532 (3)	C31—H31A	0.9900
C21—C29	1.54 (3)	C31—H31B	0.9900
C21—C25	1.547 (2)	O32—C33	1.432 (3)
C21—C28	1.55 (2)	C33—H33A	0.9800
C22—O22	1.435 (2)	C33—H33B	0.9800
C22—H22A	0.9900	C33—H33C	0.9800
C22—H22B	0.9900		
			
O22—Tb1—O24	72.47 (5)	C24—C21—C25	105.80 (15)
O22—Tb1—O14	77.75 (5)	C22—C21—C25	109.09 (15)
O24—Tb1—O14	133.94 (5)	C29—C21—C25	101.4 (11)
O22—Tb1—O12	136.11 (5)	C27—C21—C28	114.0 (14)
O24—Tb1—O12	147.08 (5)	C29—C21—C28	111.8 (16)
O14—Tb1—O12	76.53 (5)	C25—C21—C28	106.2 (9)
O22—Tb1—O13	71.20 (5)	O22—C22—C21	110.57 (16)
O24—Tb1—O13	128.37 (5)	O22—C22—H22A	109.5
O14—Tb1—O13	70.11 (5)	C21—C22—H22A	109.5
O12—Tb1—O13	66.85 (5)	O22—C22—H22B	109.5
O22—Tb1—O23	70.76 (5)	C21—C22—H22B	109.5
O24—Tb1—O23	67.19 (5)	H22A—C22—H22B	108.1
O14—Tb1—O23	70.24 (5)	O23—C23—C21	110.69 (16)
O12—Tb1—O23	130.08 (5)	O23—C23—H23A	109.5
O13—Tb1—O23	129.41 (5)	C21—C23—H23A	109.5
O22—Tb1—O4	122.01 (4)	O23—C23—H23B	109.5
O24—Tb1—O4	77.30 (5)	C21—C23—H23B	109.5
O14—Tb1—O4	148.71 (4)	H23A—C23—H23B	108.1
O12—Tb1—O4	72.71 (5)	O24—C24—C21	111.00 (16)
O13—Tb1—O4	92.32 (5)	O24—C24—H24A	109.4
O23—Tb1—O4	136.38 (5)	C21—C24—H24A	109.4
O22—Tb1—O2	142.88 (5)	O24—C24—H24B	109.4
O24—Tb1—O2	73.14 (4)	C21—C24—H24B	109.4
O14—Tb1—O2	118.55 (4)	H24A—C24—H24B	108.0
O12—Tb1—O2	80.97 (5)	C22—O22—Tb1	130.93 (12)
O13—Tb1—O2	144.10 (5)	C27—O22—Tb1	130.6 (10)
O23—Tb1—O2	83.39 (4)	C22—O22—H22O	107.2 (19)
O4—Tb1—O2	62.06 (4)	C27—O22—H22O	109 (2)
O22—Tb1—O1	130.92 (4)	Tb1—O22—H22O	119.6 (19)
O24—Tb1—O1	106.31 (4)	C23—O23—Tb1	128.64 (12)
O14—Tb1—O1	69.06 (4)	C28—O23—Tb1	130.5 (9)
O12—Tb1—O1	69.27 (4)	C23—O23—H23O	105.3 (19)
O13—Tb1—O1	125.17 (4)	C28—O23—H23O	107 (2)
O23—Tb1—O1	64.60 (4)	Tb1—O23—H23O	122.2 (19)
O4—Tb1—O1	104.45 (4)	C24—O24—Tb1	126.61 (12)
O2—Tb1—O1	49.50 (4)	C29—O24—Tb1	133.5 (12)
O22—Tb1—O5	73.45 (4)	C24—O24—H24O	108 (2)
O24—Tb1—O5	71.11 (5)	C29—O24—H24O	108 (2)
O14—Tb1—O5	131.61 (5)	Tb1—O24—H24O	118 (2)
O12—Tb1—O5	98.74 (5)	C26—C25—C21	116.65 (16)
O13—Tb1—O5	64.19 (5)	C26—C25—H25A	108.1
O23—Tb1—O5	131.18 (4)	C21—C25—H25A	108.1
O4—Tb1—O5	50.12 (4)	C26—C25—H25B	108.1
O2—Tb1—O5	107.78 (4)	C21—C25—H25B	108.1
O1—Tb1—O5	154.57 (4)	H25A—C25—H25B	107.3
C14—C11—C12	112.20 (16)	C25—C26—H26A	109.5
C14—C11—C13	111.26 (15)	C25—C26—H26B	109.5
C12—C11—C13	112.19 (15)	H26A—C26—H26B	109.5
C14—C11—C15	108.07 (15)	C25—C26—H26C	109.5
C12—C11—C15	108.42 (15)	H26A—C26—H26C	109.5
C13—C11—C15	104.27 (15)	H26B—C26—H26C	109.5
O12—C12—C11	112.61 (15)	O22—C27—C21	111.7 (16)
O12—C12—H12A	109.1	O22—C27—H27A	109.3
C11—C12—H12A	109.1	C21—C27—H27A	109.3
O12—C12—H12B	109.1	O22—C27—H27B	109.3
C11—C12—H12B	109.1	C21—C27—H27B	109.3
H12A—C12—H12B	107.8	H27A—C27—H27B	107.9
C12—O12—Tb1	130.84 (11)	O23—C28—C21	104.6 (15)
C12—O12—H12O	107.4 (19)	O23—C28—H28A	110.8
Tb1—O12—H12O	120.2 (19)	C21—C28—H28A	110.8
O13—C13—C11	114.46 (15)	O23—C28—H28B	110.8
O13—C13—H13A	108.6	C21—C28—H28B	110.8
C11—C13—H13A	108.6	H28A—C28—H28B	108.9
O13—C13—H13B	108.6	O24—C29—C21	106.4 (19)
C11—C13—H13B	108.6	O24—C29—H29A	110.5
H13A—C13—H13B	107.6	C21—C29—H29A	110.5
C13—O13—Tb1	127.60 (11)	O24—C29—H29B	110.5
C13—O13—H13O	104.1 (19)	C21—C29—H29B	110.5
Tb1—O13—H13O	113.5 (19)	H29A—C29—H29B	108.6
O14—C14—C11	110.51 (15)	O6—N2—O5	123.36 (17)
O14—C14—H14A	109.5	O6—N2—O4	121.15 (16)
C11—C14—H14A	109.5	O5—N2—O4	115.50 (15)
O14—C14—H14B	109.5	N2—O4—Tb1	98.93 (10)
C11—C14—H14B	109.5	N2—O5—Tb1	95.35 (10)
H14A—C14—H14B	108.1	O3—N1—O2	122.66 (16)
C14—O14—Tb1	131.34 (11)	O3—N1—O1	122.51 (16)
C14—O14—H14O	103.8 (19)	O2—N1—O1	114.82 (15)
Tb1—O14—H14O	117 (2)	N1—O1—Tb1	97.15 (10)
C16—C15—C11	116.62 (16)	N1—O2—Tb1	98.53 (10)
C16—C15—H15A	108.1	O7—N3—O8	120.91 (16)
C11—C15—H15A	108.1	O7—N3—O9	119.83 (16)
C16—C15—H15B	108.1	O8—N3—O9	119.26 (16)
C11—C15—H15B	108.1	O32—C31—C31^i^	111.02 (16)
H15A—C15—H15B	107.3	O32—C31—H31A	109.4
C15—C16—H16A	109.5	C31^i^—C31—H31A	109.4
C15—C16—H16B	109.5	O32—C31—H31B	109.4
H16A—C16—H16B	109.5	C31^i^—C31—H31B	109.4
C15—C16—H16C	109.5	H31A—C31—H31B	108.0
H16A—C16—H16C	109.5	C31—O32—C33	110.89 (17)
H16B—C16—H16C	109.5	O32—C33—H33A	109.5
C23—C21—C24	110.75 (17)	O32—C33—H33B	109.5
C23—C21—C22	111.65 (17)	H33A—C33—H33B	109.5
C24—C21—C22	110.43 (17)	O32—C33—H33C	109.5
C27—C21—C29	115.7 (16)	H33A—C33—H33C	109.5
C27—C21—C25	106.3 (9)	H33B—C33—H33C	109.5
C23—C21—C25	108.92 (15)		
			
C14—C11—C12—O12	−68.7 (2)	C27—C21—C25—C26	77.9 (14)
C13—C11—C12—O12	57.4 (2)	C23—C21—C25—C26	−69.4 (2)
C15—C11—C12—O12	172.01 (15)	C24—C21—C25—C26	171.49 (18)
C11—C12—O12—Tb1	10.5 (2)	C22—C21—C25—C26	52.7 (2)
C14—C11—C13—O13	73.16 (19)	C29—C21—C25—C26	−160.8 (16)
C12—C11—C13—O13	−53.5 (2)	C28—C21—C25—C26	−43.9 (12)
C15—C11—C13—O13	−170.58 (15)	Tb1—O22—C27—C21	−27 (3)
C11—C13—O13—Tb1	−18.1 (2)	C29—C21—C27—O22	78 (2)
C12—C11—C14—O14	76.17 (19)	C25—C21—C27—O22	−170.9 (15)
C13—C11—C14—O14	−50.5 (2)	C28—C21—C27—O22	−54 (2)
C15—C11—C14—O14	−164.36 (15)	Tb1—O23—C28—C21	−29 (2)
C11—C14—O14—Tb1	−25.1 (2)	C27—C21—C28—O23	78.9 (19)
C14—C11—C15—C16	−57.1 (2)	C29—C21—C28—O23	−54.7 (19)
C12—C11—C15—C16	64.8 (2)	C25—C21—C28—O23	−164.4 (12)
C13—C11—C15—C16	−175.52 (17)	Tb1—O24—C29—C21	−21 (3)
C23—C21—C22—O22	−80.2 (2)	C27—C21—C29—O24	−52 (2)
C24—C21—C22—O22	43.5 (2)	C25—C21—C29—O24	−166.2 (17)
C25—C21—C22—O22	159.38 (18)	C28—C21—C29—O24	81 (2)
C24—C21—C23—O23	−75.7 (2)	O6—N2—O4—Tb1	−177.24 (14)
C22—C21—C23—O23	47.8 (2)	O5—N2—O4—Tb1	3.28 (16)
C25—C21—C23—O23	168.37 (19)	O6—N2—O5—Tb1	177.39 (15)
C23—C21—C24—O24	42.0 (2)	O4—N2—O5—Tb1	−3.14 (15)
C22—C21—C24—O24	−82.2 (2)	O3—N1—O1—Tb1	179.72 (15)
C25—C21—C24—O24	159.84 (18)	O2—N1—O1—Tb1	0.20 (15)
C21—C22—O22—Tb1	32.0 (3)	O3—N1—O2—Tb1	−179.72 (15)
C21—C23—O23—Tb1	26.9 (3)	O1—N1—O2—Tb1	−0.21 (15)
C21—C24—O24—Tb1	42.4 (3)	C31^i^—C31—O32—C33	−174.3 (2)

Symmetry code: (i) −*x*+1/2, *y*, −*z*+1.

### (Compound-1) Dinitratobis[1,1,1-tris(hydroxymethyl)propane]terbium(III) nitrate dimethoxyethane hemisolvate Hydrogen-bond geometry (Å, º)

**Table:**

*D*—H···*A*	*D*—H	H···*A*	*D*···*A*	*D*—H···*A*
O12—H12*O*···O9^ii^	0.72 (2)	1.98 (2)	2.683 (2)	167 (3)
O13—H13*O*···O32	0.74 (2)	2.04 (2)	2.774 (2)	170 (3)
O14—H14*O*···O2^iii^	0.74 (2)	2.07 (3)	2.7935 (19)	165 (3)
O22—H22*O*···O8	0.73 (2)	2.01 (3)	2.735 (2)	172 (3)
O23—H23*O*···O4^iii^	0.69 (2)	2.16 (2)	2.8550 (19)	175 (2)
O24—H24*O*···O1^iv^	0.74 (3)	1.93 (3)	2.6624 (19)	171 (3)
C22—H22*B*···O3^v^	0.99	2.44	3.358 (3)	153
C24—H24*B*···O7^vi^	0.99	2.49	3.220 (3)	130
C29—H29*A*···O7^vi^	0.99	2.41	3.27 (3)	146

Symmetry codes: (ii) *x*+1/2, −*y*+1, *z*; (iii) −*x*+1, *y*−1/2, −*z*+3/2; (iv) −*x*+1, *y*+1/2, −*z*+3/2; (v) *x*−1/2, −*y*+1, *z*; (vi) −*x*+1/2, −*y*+3/2, −*z*+3/2.

### (Compound-2) Aquanitratobis[1,1,1-tris(hydroxymethyl)propane]terbium(III) dinitrate Crystal data

**Table:**

[Tb(NO_3_)(C_6_H_14_O_3_)_2_(H_2_O)](NO_3_)_2_	*F*(000) = 1264
*M**_r_* = 631.31	*D*_x_ = 1.858 Mg m^−^^3^
Monoclinic, *P*2_1_/*c*	Mo *K*α radiation, λ = 0.71073 Å
*a* = 9.1440 (6) Å	Cell parameters from 9581 reflections
*b* = 12.7870 (7) Å	θ = 3.1–28.3°
*c* = 19.7151 (12) Å	µ = 3.22 mm^−^^1^
β = 101.796 (2)°	*T* = 100 K
*V* = 2256.5 (2) Å^3^	Block, colourless
*Z* = 4	0.36 × 0.19 × 0.18 mm

### (Compound-2) Aquanitratobis[1,1,1-tris(hydroxymethyl)propane]terbium(III) dinitrate Data collection

**Table:**

Bruker D8 VENTURE/PHOTON100 CMOS diffractometer	5629 independent reflections
Radiation source: fine-focus sealed tube	5542 reflections with *I* > 2σ(*I*)
Graphite monochromator	*R*_int_ = 0.023
Detector resolution: 10.4167 pixels mm^-1^	θ_max_ = 28.4°, θ_min_ = 3.6°
φ and ω scans	*h* = −12→12
Absorption correction: multi-scan (SADABS; Bruker, 2014)	*k* = −17→17
*T*_min_ = 0.636, *T*_max_ = 0.746	*l* = −26→26
244328 measured reflections	

### (Compound-2) Aquanitratobis[1,1,1-tris(hydroxymethyl)propane]terbium(III) dinitrate Refinement

**Table:**

Refinement on *F*^2^	Primary atom site location: structure-invariant direct methods
Least-squares matrix: full	Hydrogen site location: mixed
*R*[*F*^2^ > 2σ(*F*^2^)] = 0.012	H atoms treated by a mixture of independent and constrained refinement
*wR*(*F*^2^) = 0.029	*w* = 1/[σ^2^(*F*_o_^2^) + (0.0128*P*)^2^ + 1.457*P*] where *P* = (*F*_o_^2^ + 2*F*_c_^2^)/3
*S* = 1.13	(Δ/σ)_max_ = 0.001
5629 reflections	Δρ_max_ = 0.74 e Å^−^^3^
326 parameters	Δρ_min_ = −0.26 e Å^−^^3^
0 restraints	

### (Compound-2) Aquanitratobis[1,1,1-tris(hydroxymethyl)propane]terbium(III) dinitrate Special details

**Table:**

Geometry. All esds (except the esd in the dihedral angle between two l.s. planes) are estimated using the full covariance matrix. The cell esds are taken into account individually in the estimation of esds in distances, angles and torsion angles; correlations between esds in cell parameters are only used when they are defined by crystal symmetry. An approximate (isotropic) treatment of cell esds is used for estimating esds involving l.s. planes.

### (Compound-2) Aquanitratobis[1,1,1-tris(hydroxymethyl)propane]terbium(III) dinitrate Fractional atomic coordinates and isotropic or equivalent isotropic displacement parameters (Å^2^)

**Table:**

	*x*	*y*	*z*	*U*_iso_*/*U*_eq_	Occ. (<1)
Tb1	0.15357 (2)	0.39026 (2)	0.37356 (2)	0.00991 (2)	
C11	0.20317 (14)	0.11240 (9)	0.41042 (7)	0.0134 (2)	
C12	0.05513 (15)	0.14541 (10)	0.42837 (7)	0.0170 (2)	
H12A	−0.0282	0.1120	0.3955	0.020*	
H12B	0.0516	0.1206	0.4756	0.020*	
O12	0.03581 (10)	0.25678 (7)	0.42542 (5)	0.01517 (17)	
C13	0.19968 (16)	0.12622 (10)	0.33317 (7)	0.0168 (2)	
H13A	0.3014	0.1158	0.3243	0.020*	
H13B	0.1335	0.0726	0.3067	0.020*	
O13	0.14671 (11)	0.22883 (7)	0.30972 (5)	0.01632 (18)	
C14	0.33441 (14)	0.17092 (10)	0.45474 (7)	0.0159 (2)	
H14A	0.3238	0.1703	0.5037	0.019*	
H14B	0.4288	0.1347	0.4521	0.019*	
O14	0.34140 (11)	0.27755 (7)	0.43195 (5)	0.01548 (18)	
C15	0.22392 (16)	−0.00526 (10)	0.42866 (7)	0.0191 (3)	
H15A	0.2419	−0.0124	0.4797	0.023*	0.827 (4)
H15B	0.1291	−0.0418	0.4093	0.023*	0.827 (4)
H15C	0.2488	−0.0122	0.4797	0.023*	0.173 (4)
H15D	0.3105	−0.0315	0.4106	0.023*	0.173 (4)
C16	0.3507 (2)	−0.06169 (13)	0.40298 (9)	0.0230 (4)	0.827 (4)
H16A	0.3537	−0.1352	0.4173	0.034*	0.827 (4)
H16B	0.3331	−0.0577	0.3523	0.034*	0.827 (4)
H16C	0.4462	−0.0282	0.4229	0.034*	0.827 (4)
C17	0.0871 (9)	−0.0763 (6)	0.4002 (4)	0.0184 (19)*	0.173 (4)
H17A	0.1111	−0.1488	0.4144	0.028*	0.173 (4)
H17B	0.0011	−0.0528	0.4188	0.028*	0.173 (4)
H17C	0.0629	−0.0722	0.3495	0.028*	0.173 (4)
C21	0.36977 (14)	0.59444 (10)	0.30982 (7)	0.0141 (2)	
C22	0.32638 (15)	0.51321 (10)	0.25283 (6)	0.0157 (2)	
H22A	0.2465	0.5419	0.2159	0.019*	
H22B	0.4139	0.4975	0.2321	0.019*	
O22	0.27453 (11)	0.41792 (7)	0.27942 (5)	0.01562 (17)	
C23	0.46779 (14)	0.54531 (10)	0.37450 (7)	0.0158 (2)	
H23A	0.5405	0.4972	0.3600	0.019*	
H23B	0.5249	0.6013	0.4030	0.019*	
O23	0.38207 (10)	0.48831 (7)	0.41624 (5)	0.01434 (17)	
C24	0.23305 (14)	0.64798 (10)	0.32772 (7)	0.0157 (2)	
H24A	0.2650	0.6928	0.3690	0.019*	
H24B	0.1856	0.6933	0.2887	0.019*	
O24	0.12570 (11)	0.57223 (7)	0.34145 (5)	0.01533 (17)	
C25	0.46674 (15)	0.67891 (11)	0.28378 (7)	0.0194 (3)	
H25A	0.4749	0.7398	0.3154	0.023*	
H25B	0.5686	0.6503	0.2872	0.023*	
C26	0.41016 (18)	0.71744 (12)	0.20979 (8)	0.0256 (3)	
H26A	0.4789	0.7703	0.1984	0.038*	
H26B	0.4046	0.6585	0.1776	0.038*	
H26C	0.3106	0.7482	0.2058	0.038*	
N1	−0.15472 (12)	0.43478 (8)	0.30929 (6)	0.0147 (2)	
O1	−0.06405 (11)	0.39309 (7)	0.27587 (5)	0.01717 (18)	
O2	−0.10340 (10)	0.45124 (7)	0.37367 (5)	0.01666 (18)	
O3	−0.28209 (11)	0.45781 (8)	0.28147 (5)	0.0226 (2)	
N2	0.72651 (12)	0.29286 (8)	0.48389 (5)	0.01374 (19)	
O4	0.70888 (11)	0.20729 (8)	0.45553 (5)	0.0224 (2)	
O5	0.85643 (10)	0.32966 (7)	0.50584 (5)	0.01657 (18)	
O6	0.61580 (10)	0.34701 (8)	0.49253 (5)	0.0213 (2)	
N3	0.18037 (13)	0.24790 (9)	0.13412 (6)	0.0163 (2)	
O7	0.19294 (13)	0.21012 (8)	0.07818 (5)	0.0253 (2)	
O8	0.10941 (12)	0.19955 (8)	0.17319 (5)	0.0226 (2)	
O9	0.24079 (15)	0.33293 (9)	0.15397 (6)	0.0319 (3)	
O10	0.15368 (11)	0.45418 (8)	0.48688 (5)	0.01625 (18)	
H1OA	0.160 (2)	0.4204 (17)	0.5185 (11)	0.032 (5)*	
H1OB	0.148 (2)	0.5141 (18)	0.4948 (11)	0.036 (6)*	
H12O	−0.017 (2)	0.2727 (15)	0.4478 (10)	0.028 (5)*	
H13O	0.131 (2)	0.2279 (15)	0.2698 (11)	0.027 (5)*	
H14O	0.416 (2)	0.2983 (16)	0.4460 (10)	0.030 (5)*	
H22O	0.255 (2)	0.3813 (16)	0.2486 (12)	0.034 (6)*	
H23O	0.371 (2)	0.5220 (16)	0.4430 (10)	0.026 (5)*	
H24O	0.058 (2)	0.6003 (15)	0.3462 (10)	0.023 (5)*	

### (Compound-2) Aquanitratobis[1,1,1-tris(hydroxymethyl)propane]terbium(III) dinitrate Atomic displacement parameters (Å^2^)

**Table:**

	*U*^11^	*U*^22^	*U*^33^	*U*^12^	*U*^13^	*U*^23^
Tb1	0.01123 (3)	0.00934 (3)	0.00935 (3)	0.00074 (2)	0.00253 (2)	−0.00031 (2)
C11	0.0172 (6)	0.0099 (5)	0.0137 (6)	0.0001 (4)	0.0042 (4)	−0.0001 (4)
C12	0.0188 (6)	0.0110 (6)	0.0231 (6)	−0.0014 (5)	0.0088 (5)	0.0006 (5)
O12	0.0168 (4)	0.0119 (4)	0.0195 (4)	0.0010 (3)	0.0102 (4)	−0.0010 (3)
C13	0.0243 (6)	0.0118 (5)	0.0148 (6)	0.0030 (5)	0.0053 (5)	−0.0012 (4)
O13	0.0262 (5)	0.0123 (4)	0.0100 (4)	0.0026 (4)	0.0026 (4)	−0.0008 (3)
C14	0.0176 (6)	0.0114 (5)	0.0175 (6)	0.0017 (5)	0.0007 (5)	0.0019 (5)
O14	0.0128 (4)	0.0110 (4)	0.0206 (5)	−0.0008 (3)	−0.0014 (3)	0.0007 (3)
C15	0.0251 (7)	0.0113 (6)	0.0213 (6)	0.0012 (5)	0.0052 (5)	0.0018 (5)
C16	0.0315 (9)	0.0147 (8)	0.0227 (8)	0.0072 (6)	0.0054 (7)	0.0015 (6)
C21	0.0153 (6)	0.0127 (5)	0.0155 (6)	−0.0008 (4)	0.0058 (4)	−0.0002 (4)
C22	0.0202 (6)	0.0136 (6)	0.0146 (5)	−0.0023 (5)	0.0067 (5)	0.0007 (4)
O22	0.0215 (5)	0.0119 (4)	0.0154 (4)	−0.0024 (4)	0.0082 (4)	−0.0030 (4)
C23	0.0136 (5)	0.0176 (6)	0.0170 (6)	−0.0018 (5)	0.0050 (4)	−0.0002 (5)
O23	0.0160 (4)	0.0138 (4)	0.0135 (4)	−0.0010 (3)	0.0039 (3)	−0.0010 (3)
C24	0.0176 (6)	0.0111 (5)	0.0199 (6)	−0.0003 (4)	0.0072 (5)	0.0008 (5)
O24	0.0139 (4)	0.0117 (4)	0.0223 (5)	0.0020 (3)	0.0081 (4)	0.0011 (3)
C25	0.0215 (6)	0.0156 (6)	0.0235 (6)	−0.0038 (5)	0.0101 (5)	0.0002 (5)
C26	0.0306 (7)	0.0204 (7)	0.0286 (7)	−0.0004 (6)	0.0126 (6)	0.0078 (6)
N1	0.0141 (5)	0.0139 (5)	0.0162 (5)	0.0002 (4)	0.0036 (4)	0.0037 (4)
O1	0.0157 (4)	0.0220 (5)	0.0142 (4)	0.0024 (3)	0.0037 (3)	−0.0023 (3)
O2	0.0181 (4)	0.0202 (5)	0.0123 (4)	0.0027 (4)	0.0046 (3)	0.0013 (3)
O3	0.0132 (4)	0.0289 (5)	0.0247 (5)	0.0042 (4)	0.0014 (4)	0.0064 (4)
N2	0.0149 (5)	0.0149 (5)	0.0119 (5)	0.0005 (4)	0.0037 (4)	0.0002 (4)
O4	0.0239 (5)	0.0153 (4)	0.0289 (5)	−0.0022 (4)	0.0075 (4)	−0.0079 (4)
O5	0.0128 (4)	0.0182 (4)	0.0193 (4)	−0.0013 (3)	0.0046 (3)	−0.0026 (4)
O6	0.0125 (4)	0.0218 (5)	0.0287 (5)	0.0025 (4)	0.0023 (4)	−0.0096 (4)
N3	0.0200 (5)	0.0160 (5)	0.0130 (5)	−0.0023 (4)	0.0036 (4)	−0.0019 (4)
O7	0.0388 (6)	0.0249 (5)	0.0143 (4)	−0.0077 (4)	0.0104 (4)	−0.0073 (4)
O8	0.0263 (5)	0.0272 (5)	0.0158 (4)	−0.0126 (4)	0.0079 (4)	−0.0039 (4)
O9	0.0526 (7)	0.0215 (5)	0.0251 (5)	−0.0174 (5)	0.0161 (5)	−0.0096 (4)
O10	0.0251 (5)	0.0129 (4)	0.0114 (4)	0.0005 (4)	0.0054 (4)	−0.0003 (4)

### (Compound-2) Aquanitratobis[1,1,1-tris(hydroxymethyl)propane]terbium(III) dinitrate Geometric parameters (Å, º)

**Table:**

Tb1—O14	2.3545 (9)	C17—H17B	0.9800
Tb1—O12	2.3597 (9)	C17—H17C	0.9800
Tb1—O22	2.3734 (9)	C21—C22	1.5220 (17)
Tb1—O10	2.3786 (9)	C21—C24	1.5286 (17)
Tb1—O24	2.4112 (9)	C21—C23	1.5353 (18)
Tb1—O13	2.4119 (9)	C21—C25	1.5499 (17)
Tb1—O23	2.4344 (9)	C22—O22	1.4443 (15)
Tb1—O1	2.4706 (10)	C22—H22A	0.9900
Tb1—O2	2.4762 (9)	C22—H22B	0.9900
C11—C13	1.5269 (18)	O22—H22O	0.76 (2)
C11—C12	1.5271 (18)	C23—O23	1.4444 (15)
C11—C14	1.5281 (17)	C23—H23A	0.9900
C11—C15	1.5497 (17)	C23—H23B	0.9900
C12—O12	1.4347 (15)	O23—H23O	0.71 (2)
C12—H12A	0.9900	C24—O24	1.4436 (15)
C12—H12B	0.9900	C24—H24A	0.9900
O12—H12O	0.74 (2)	C24—H24B	0.9900
C13—O13	1.4417 (15)	O24—H24O	0.73 (2)
C13—H13A	0.9900	C25—C26	1.526 (2)
C13—H13B	0.9900	C25—H25A	0.9900
O13—H13O	0.77 (2)	C25—H25B	0.9900
C14—O14	1.4412 (15)	C26—H26A	0.9800
C14—H14A	0.9900	C26—H26B	0.9800
C14—H14B	0.9900	C26—H26C	0.9800
O14—H14O	0.73 (2)	N1—O3	1.2177 (14)
C15—C16	1.537 (2)	N1—O1	1.2764 (14)
C15—C17	1.555 (8)	N1—O2	1.2775 (14)
C15—H15A	0.9900	N2—O4	1.2244 (15)
C15—H15B	0.9900	N2—O6	1.2664 (14)
C15—H15C	0.9900	N2—O5	1.2689 (14)
C15—H15D	0.9900	N3—O7	1.2308 (14)
C16—H16A	0.9800	N3—O9	1.2458 (15)
C16—H16B	0.9800	N3—O8	1.2652 (14)
C16—H16C	0.9800	O10—H1OA	0.75 (2)
C17—H17A	0.9800	O10—H1OB	0.79 (2)
			
O14—Tb1—O12	72.38 (3)	C11—C15—H15D	108.4
O14—Tb1—O22	93.64 (3)	C17—C15—H15D	108.4
O12—Tb1—O22	140.67 (3)	H15C—C15—H15D	107.4
O14—Tb1—O10	83.74 (3)	C15—C16—H16A	109.5
O12—Tb1—O10	75.77 (3)	C15—C16—H16B	109.5
O22—Tb1—O10	140.58 (3)	H16A—C16—H16B	109.5
O14—Tb1—O24	138.28 (3)	C15—C16—H16C	109.5
O12—Tb1—O24	141.14 (3)	H16A—C16—H16C	109.5
O22—Tb1—O24	72.06 (3)	H16B—C16—H16C	109.5
O10—Tb1—O24	83.96 (3)	C15—C17—H17A	109.5
O14—Tb1—O13	70.72 (3)	C15—C17—H17B	109.5
O12—Tb1—O13	68.99 (3)	H17A—C17—H17B	109.5
O22—Tb1—O13	71.69 (3)	C15—C17—H17C	109.5
O10—Tb1—O13	141.19 (3)	H17A—C17—H17C	109.5
O24—Tb1—O13	134.33 (3)	H17B—C17—H17C	109.5
O14—Tb1—O23	69.97 (3)	C22—C21—C24	111.96 (11)
O12—Tb1—O23	131.17 (3)	C22—C21—C23	110.72 (10)
O22—Tb1—O23	71.90 (3)	C24—C21—C23	110.95 (10)
O10—Tb1—O23	70.30 (3)	C22—C21—C25	108.15 (10)
O24—Tb1—O23	68.34 (3)	C24—C21—C25	108.34 (10)
O13—Tb1—O23	123.37 (3)	C23—C21—C25	106.50 (10)
O14—Tb1—O1	141.34 (3)	O22—C22—C21	111.30 (10)
O12—Tb1—O1	88.98 (3)	O22—C22—H22A	109.4
O22—Tb1—O1	79.51 (3)	C21—C22—H22A	109.4
O10—Tb1—O1	124.95 (3)	O22—C22—H22B	109.4
O24—Tb1—O1	75.88 (3)	C21—C22—H22B	109.4
O13—Tb1—O1	71.02 (3)	H22A—C22—H22B	108.0
O23—Tb1—O1	139.42 (3)	C22—O22—Tb1	130.51 (7)
O14—Tb1—O2	142.17 (3)	C22—O22—H22O	105.7 (16)
O12—Tb1—O2	73.16 (3)	Tb1—O22—H22O	117.7 (16)
O22—Tb1—O2	123.21 (3)	O23—C23—C21	112.77 (10)
O10—Tb1—O2	73.09 (3)	O23—C23—H23A	109.0
O24—Tb1—O2	69.33 (3)	C21—C23—H23A	109.0
O13—Tb1—O2	110.27 (3)	O23—C23—H23B	109.0
O23—Tb1—O2	125.81 (3)	C21—C23—H23B	109.0
O1—Tb1—O2	51.89 (3)	H23A—C23—H23B	107.8
C13—C11—C12	111.13 (11)	C23—O23—Tb1	126.18 (7)
C13—C11—C14	111.57 (10)	C23—O23—H23O	107.4 (16)
C12—C11—C14	111.21 (10)	Tb1—O23—H23O	109.0 (16)
C13—C11—C15	108.69 (10)	O24—C24—C21	111.24 (10)
C12—C11—C15	106.67 (10)	O24—C24—H24A	109.4
C14—C11—C15	107.34 (10)	C21—C24—H24A	109.4
O12—C12—C11	111.87 (10)	O24—C24—H24B	109.4
O12—C12—H12A	109.2	C21—C24—H24B	109.4
C11—C12—H12A	109.2	H24A—C24—H24B	108.0
O12—C12—H12B	109.2	C24—O24—Tb1	131.10 (8)
C11—C12—H12B	109.2	C24—O24—H24O	108.4 (15)
H12A—C12—H12B	107.9	Tb1—O24—H24O	118.9 (15)
C12—O12—Tb1	132.24 (8)	C26—C25—C21	115.88 (12)
C12—O12—H12O	109.7 (15)	C26—C25—H25A	108.3
Tb1—O12—H12O	117.7 (15)	C21—C25—H25A	108.3
O13—C13—C11	111.25 (10)	C26—C25—H25B	108.3
O13—C13—H13A	109.4	C21—C25—H25B	108.3
C11—C13—H13A	109.4	H25A—C25—H25B	107.4
O13—C13—H13B	109.4	C25—C26—H26A	109.5
C11—C13—H13B	109.4	C25—C26—H26B	109.5
H13A—C13—H13B	108.0	H26A—C26—H26B	109.5
C13—O13—Tb1	129.81 (7)	C25—C26—H26C	109.5
C13—O13—H13O	107.0 (15)	H26A—C26—H26C	109.5
Tb1—O13—H13O	121.7 (15)	H26B—C26—H26C	109.5
O14—C14—C11	111.38 (10)	O3—N1—O1	122.06 (11)
O14—C14—H14A	109.4	O3—N1—O2	122.07 (11)
C11—C14—H14A	109.4	O1—N1—O2	115.87 (10)
O14—C14—H14B	109.4	O3—N1—Tb1	177.09 (9)
C11—C14—H14B	109.4	O1—N1—Tb1	57.85 (6)
H14A—C14—H14B	108.0	O2—N1—Tb1	58.10 (6)
C14—O14—Tb1	131.09 (8)	N1—O1—Tb1	96.21 (7)
C14—O14—H14O	108.8 (16)	N1—O2—Tb1	95.92 (7)
Tb1—O14—H14O	119.6 (16)	O4—N2—O6	121.06 (11)
C16—C15—C11	116.37 (12)	O4—N2—O5	120.92 (11)
C11—C15—C17	115.6 (3)	O6—N2—O5	118.02 (10)
C16—C15—H15A	108.2	O7—N3—O9	120.49 (11)
C11—C15—H15A	108.2	O7—N3—O8	120.47 (11)
C16—C15—H15B	108.2	O9—N3—O8	119.01 (11)
C11—C15—H15B	108.2	Tb1—O10—H1OA	124.7 (16)
H15A—C15—H15B	107.3	Tb1—O10—H1OB	122.4 (15)
C11—C15—H15C	108.4	H1OA—O10—H1OB	113 (2)
C17—C15—H15C	108.4		
			
C13—C11—C12—O12	−72.04 (13)	C23—C21—C22—O22	46.85 (14)
C14—C11—C12—O12	52.90 (14)	C25—C21—C22—O22	163.18 (10)
C15—C11—C12—O12	169.64 (10)	C21—C22—O22—Tb1	29.13 (15)
C11—C12—O12—Tb1	17.38 (16)	C22—C21—C23—O23	−79.94 (13)
C12—C11—C13—O13	49.60 (14)	C24—C21—C23—O23	45.02 (14)
C14—C11—C13—O13	−75.14 (13)	C25—C21—C23—O23	162.72 (10)
C15—C11—C13—O13	166.69 (11)	C21—C23—O23—Tb1	33.74 (14)
C11—C13—O13—Tb1	25.43 (15)	C22—C21—C24—O24	51.36 (14)
C13—C11—C14—O14	49.60 (14)	C23—C21—C24—O24	−72.89 (13)
C12—C11—C14—O14	−75.10 (13)	C25—C21—C24—O24	170.54 (10)
C15—C11—C14—O14	168.57 (10)	C21—C24—O24—Tb1	20.64 (15)
C11—C14—O14—Tb1	26.41 (15)	C22—C21—C25—C26	44.73 (15)
C13—C11—C15—C16	49.30 (16)	C24—C21—C25—C26	−76.83 (14)
C12—C11—C15—C16	169.20 (12)	C23—C21—C25—C26	163.76 (11)
C14—C11—C15—C16	−71.51 (15)	O3—N1—O1—Tb1	176.57 (10)
C13—C11—C15—C17	−70.7 (3)	O2—N1—O1—Tb1	−3.17 (11)
C12—C11—C15—C17	49.2 (3)	O3—N1—O2—Tb1	−176.58 (10)
C14—C11—C15—C17	168.5 (3)	O1—N1—O2—Tb1	3.16 (11)
C24—C21—C22—O22	−77.54 (13)		

### (Compound-2) Aquanitratobis[1,1,1-tris(hydroxymethyl)propane]terbium(III) dinitrate Hydrogen-bond geometry (Å, º)

**Table:**

*D*—H···*A*	*D*—H	H···*A*	*D*···*A*	*D*—H···*A*
O10—H1*OA*···O7^i^	0.75 (2)	2.03 (2)	2.7420 (14)	159 (2)
O10—H1*OB*···O5^ii^	0.79 (2)	2.00 (2)	2.7703 (14)	167 (2)
O13—H13*O*···O8	0.77 (2)	1.91 (2)	2.6695 (14)	169 (2)
O12—H12*O*···O5^iii^	0.74 (2)	1.93 (2)	2.6713 (13)	174 (2)
O14—H14*O*···O6	0.73 (2)	1.97 (2)	2.6992 (14)	174 (2)
O23—H23*O*···O6^ii^	0.71 (2)	2.09 (2)	2.7669 (14)	161 (2)
O22—H22*O*···O9	0.76 (2)	1.94 (2)	2.6609 (14)	157 (2)
O24—H24*O*···O8^iv^	0.73 (2)	1.97 (2)	2.6650 (14)	158 (2)
C14—H14*A*···O7^i^	0.99	2.58	3.3462 (17)	135
C23—H23*A*···O3^v^	0.99	2.51	3.4003 (16)	149

Symmetry codes: (i) *x*, −*y*+1/2, *z*+1/2; (ii) −*x*+1, −*y*+1, −*z*+1; (iii) *x*−1, *y*, *z*; (iv) −*x*, *y*+1/2, −*z*+1/2; (v) *x*+1, *y*, *z*.
